# Membrane-Based Solvent Exchange Process for Purification of API Crystal Suspensions

**DOI:** 10.3390/membranes13030263

**Published:** 2023-02-23

**Authors:** Fatima Anjum, Maximilian Wessner, Gabriele Sadowski

**Affiliations:** Department of Biochemical and Chemical Engineering, Laboratory of Thermodynamics, TU Dortmund University, Emil-Figge-Str. 70, D-44227 Dortmund, Germany

**Keywords:** diafiltration, long acting injectables, pharmaceutical crystal suspensions, organic solvent nanofiltration

## Abstract

Bottom-up approaches to producing aqueous crystal suspensions of active pharmaceutical ingredients (APIs), such as anti-solvent crystallisation, are gaining interest as they offer better control over surface properties compared to top-down approaches. However, one of the major challenges that needs to be addressed is the removal of organic solvents after the crystallisation step due to strict limitations regarding human exposure. Within this work, we investigated a process concept for the removal of solvent (i.e., ethanol) from the API crystal suspension using membrane-based diafiltration. A four-stage diafiltration process successfully reduced the ethanol concentration in the API (here, naproxen) crystal suspension below 0.5 wt% (the residual solvent limit as per ICH guidelines) with a water consumption of 1.5 g of added water per g of feed. The solvent exchange process had no negative influence on the stability of the crystals in suspension, as their size and polymorphic form remained unchanged. This work is a step towards the bottom-up production of API crystal suspension by applying solvent/anti-solvent crystallisation. It provides the proof of concept for establishing a process of organic solvent removal and offers an experimental framework to serve as the foundation for the design of experiments implementing a solvent exchange in API production processes.

## 1. Introduction

Long-acting crystal suspensions (particle size < 10 μm) of active pharmaceutical ingredients (APIs) have gained popularity over common formulation strategies, offering several benefits such as: (1) solubilization of poorly water-soluble APIs; (2) higher physical stability (compared to classical formulations); (3) improved intramuscular penetration; and (4) a higher patients’ convenience [1]. In 2020, Nkanga et al. [2] reported nine marketed long-acting crystal suspensions, with Invega Sustenna^®^ and Invega Trinza^®^ from Janssen Pharmaceutica showing the highest sale of USD 3.3 billion in the year 2019. According to the Johnson and Johnson 2021 annual report [3], the sale of these drug suspensions is increasing by 10% annually, which indicates the increasing popularity and demand of these long-acting crystal suspensions.

All the marketed long-acting crystal suspensions are produced by breaking down large particles via a milling process (a top-down approach) [2]. This approach is very energy intensive, and the surface properties of the crystals cannot be controlled very well. An alternative approach is building up particles from the molecular level (a bottom-up approach) using crystallisation techniques [4], which offer better control over the surface properties of the particles. One such technique is anti-solvent crystallisation, where the API is dissolved in an organic solvent, which offers high API solubility compared to water. The latter is used as an anti-solvent. Adding this anti-solvent to the organic solution leads to exceeding the API solubility, and the API crystallizes. During crystallisation, it is important to control the particle-size growth to achieve the required particle size distribution. For this purpose, polymeric excipients are used to control the particle size and, thus, stabilise the crystal suspension [5]. However, there are some challenges that need to be addressed before the commercialization of the bottom-up approach for the production of long-acting crystal suspensions, e.g., finding the right excipient, purification of the crystal suspension, and increasing the API concentration. One major challenge is the concentration of the organic solvent in the suspension, which, directly after crystallisation, is higher than the acceptable exposure limit for human administration. Thus, the removal of the organic solvent from the final suspension is required.

According to the guidelines provided by the International Council for Harmonization (ICH), the residual amount of class three solvents (e.g., ethanol, acetone, etc.) in injections should be less than 0.5 wt% [6]. The removal of organic solvent with conventional separation techniques (e.g., evaporation, distillation, or absorption) is very costly and energy intensive or even not possible [7,8]. One promising alternative are membrane-based purification processes that offer benefits such as: (1) compliance of the ICH purity requirements; (2) non-thermal treatment, i.e., separation at ambient temperature without application of heat (as in case of evaporation or distillation); and (3) reduced energy consumption and chemical requirements compared to conventional separation techniques [9].

Membrane processes have been widely used in the pharmaceutical industry for various applications like waste-water treatment to remove toxic and hazardous components [10,11,12], solvent recovery during intermediate processing steps for recycling [8,9,13], API recovery [14], buffer exchange, protein purification, and protein concentration for biomolecule manufacturing [15,16,17]. However, the application of membranes in API crystallisation and crystal purification (especially for particle size < 10 μm) is limited. As another promising application of membranes in this field, we propose their application for solvent exchange after anti-solvent crystallisation to replace residual organic solvents with water. For that purpose, organic-solvent-nanofiltration (OSN) membranes are of particular interest due to the following reasons: (1) high stability in the presence of organic solvents and water; (2) high retention of crystals and excipients; and (3) easy integration of crystallisation and solvent-exchange processes. OSN membranes have found various applications in the pharmaceutical industries, including: solvent recovery and recycling [18,19], removal of impurities during API manufacturing [20,21], API recovery and concentration [22,23], catalyst recovery [24], and pharmaceutical wastewater treatment [25].

A diafiltration process is of particular interest for applications that require very low residual concentrations of a specific component. Diafiltration is a membrane-based filtration process that is used to remove low-molecular-weight components from a solution or suspension [26]. During this process, high-molecular-weight components are retained by the membrane while a new solvent or buffer is added to the feed to dilute/wash out the low-molecular-weight components (solvents) [27,28]. This process has already been applied for desalting [29], buffer exchange [17], protein purification [30], nanoparticle purification [31], and API molecule recovery and purification [32]. Sheth et al. [9] successfully applied diafiltration for solvent exchange during the intermediate steps of the API manufacturing process. However, their application employed a feed solution with dissolved API, but no suspended particles were present during the solvent exchange. In this work, we have now developed a process concept for the purification of API crystals suspended in mixtures of organic solvent and water.

The process design for a membrane-based solvent exchange using diafiltration comprised of the following steps:

(1) Finding a suitable membrane. For that purpose, we screened commercially available OSN membranes based on their permeate flux and separation factor. A nanofiltration membrane was used to ensure complete retention of crystals and excipients (used during crystallisation).
(2) Determining the amount of water required for solvent exchange via diafiltration. It is important to note here that for a membrane-based solvent exchange, the miscibility of the liquid phase is desired. In cases of immiscibility, the liquid-phase transport through the membrane is not homogenous and highly depends on the mixing conditions [9]. Thus, in this study, ethanol was used as an organic solvent. It is miscible with water (used as an anti-solvent and dilutant) at its operating temperature, i.e., 25 °C.
(3) Understanding different influencing factors, e.g., type of crystals, solvent concentration, and stabilizing excipients, on the permeate flux through the membrane to optimize membrane performance.
(4) Selecting the optimal mode of operation for diafiltration by comparing performance in terms of flux, water consumption, and operability during both semi-continuous and discontinuous modes.

To the best of our knowledge, the diafiltration process has not yet been applied for solvent exchange of API crystal suspensions. Thus, this work offers a complete process-design study to implement the diafiltration process for the solvent exchange in API crystal suspension and highlights key factors that need to be taken into account during process development.

## 2. Materials and Methods

### 2.1. Materials

Table 1 lists the chemicals and gases used in this study, along with their purity and supplier information. All chemicals were used as received.

Table 2 lists the OSN membranes used in this study with all the details. Membranes were supplied as dry flat sheets, except for AMS NanoPro, which was supplied wet and should not dry out. Membranes were cut into circular discs of 90 mm diameter prior to use in the membrane module.

### 2.2. Preparation of API Crystal Suspensions

Naproxen (MW 230.26 g mol^−1^) was used as a model API because naproxen properties are widely reported in the literature, which helped us in solubility modelling and in selecting process parameters. The naproxen suspension was prepared using solvent/anti-solvent crystallisation, with ethanol as the solvent and water as the anti-solvent. Ethanol was used as solvent owing to the benefits it offered for the crystallisation process, such as complete miscibility with water at operating temperature, i.e., 25 °C, reasonable solubility of naproxen (solubility of naproxen in ethanol at 25 °C: 5.9 wt% [33]), the fact that it belongs to class three solvents [6], is less toxic compared to solvents like methanol, acetonitrile, and N-Methyl 2 pyrrolidone, and is also readily available at a low price. The crystallisation set-up was built with a 3 mm (internal diameter) y-connector having a hold-up volume of 0.2 mL and connected to water and ethanol tanks with pharmaceutical-grade polypropylene-based tubing (PharMed^®^ BPT by Saint-Gobain Biopharm, Charny, France). Peristaltic pumps (pump head: Masterflex L/S Easy Load 2, drive: Ecoline, Ismatec, Wertheim, Germany) were used for the transport of water and ethanol solutions. The suspension was collected in a jacketed, stirred glass bottle maintained at 25 °C using a thermostat (ECO Silver RE 420 by Lauda, Lauda-Königshofen, Germany).

For crystallisation, ethanol with 4 wt% dissolved naproxen was used as a solvent stream, and water with 0.1 wt% dissolved HPMC E3 (an excipient) was used as an anti-solvent stream. After complete dissolution, both solutions were filtered through 0.45-μm Polytetrafluoroethylene (PTFE) filters (Whatman^®^ Gmbh, Dassel, Germany) prior to crystallisation. Crystallisation was conducted at 25 °C. The ethanol side flow rate was 12 mL min^−1^ and the water side flow rate was 112 mL min^−1^, thus the solvent to anti-solvent ratio (based on volumetric flow rates) was 1:9.3. All operating parameters and final mass fractions of the crystal suspension are provided in Table 3. For further uses, the suspension was stored at 25°C under stirred conditions.

### 2.3. Experimental Setup

**Figure 1:**
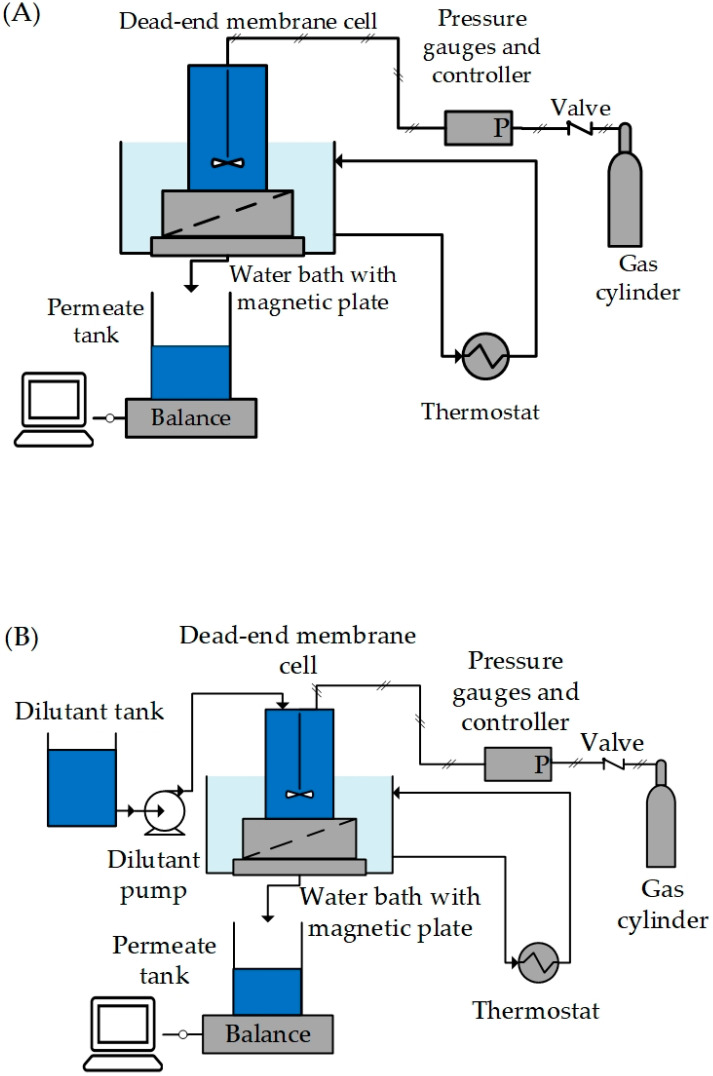
Experimental setup for membrane screening, discontinuous diafiltration (**A**), and semi-continuous diafiltration experiments (**B**). It consisted of a dead-end membrane module with an active membrane area of 54 cm^2^, placed on a magnetic stirrer in a temperature-controlled water bath.

Figure 1 shows the experimental setup used in this study.

Figure 1A shows the membrane screening and discontinuous diafiltration setup. It consisted of a dead-end membrane module supplied by Evonik MET in the United Kingdom. The membrane module is comprised of a feed tank with a maximum volume of 250 mL, a suspended magnetic stirrer attached to the top lid, and a filtration cell with an active membrane area of 54 cm^2^. The membrane module also consisted of a sintered stainless-steel disc to support the membrane and fluorinated ethylene propylene (FEP)-coated O-rings to prevent pressure loss and/or leakage. The membrane module was placed on a magnetic stirrer in a temperature-controlled water bath. Figure 1B shows the setup for semi-continuous diafiltration. It consists of an additional dilutant tank connected to the membrane module via a pump (the L-6200 Intelligent pump from Hitachi Ltd., Tokyo, Japan) for continuous addition of dilutant (here, water) during semi-continuous diafiltration.

The temperature for all experiments was maintained at 25 °C with the help of a thermostat provided by Julabo Labortechnik Gmbh, Seelbach, Germany. The membrane module was pressurised using nitrogen gas, and pressure was controlled using the gas control unit provided by Evonik MET, Greenford, United Kingdom. Permeate was collected in glass bottles and placed on a mass balance (provided by Mettler Toledo, Hessen, Germany) having a precision of ±0.01 g. The balance was connected to a computer, and mass data was continuously logged with the help of LABVIEW software (National Instruments Corp., Texas, USA). All experiments were performed at 30 bar, 25 °C and 250 revolutions per minute (rpm) of the stirrer unless otherwise stated.

### 2.4. Experimental Procedures

#### 2.4.1. Membrane Cleaning and Conditioning

For some membranes, membrane cleaning or conditioning was required prior to the experiments. Duramem membranes contained preservatives that had to be removed prior to use. The membranes were submerged in ethanol overnight, and after installation of the membrane in the membrane module, 150 mL of ethanol was permeated through the membrane to ensure complete removal of preservatives. Afterwards, the membranes were conditioned by permeating water through them. AMS membranes were supplied wet; therefore, they were washed with water after installation, and then conditioning was performed by permeating more water through the membrane as mentioned above. In the case of other membranes, no cleaning was required. Hence, they were just conditioned prior to experimentation.

#### 2.4.2. Membrane Screening

Membrane screening was done based on two performance parameters, i.e., permeate flux and separation factor (SF). The permeate flux is defined as the mass of liquid permeating through the membrane per unit area and time. Mass flow was obtained gravimetrically by measuring the change in mass of the permeating liquid over time, and mass flux was determined using the equation:

(1) $$J=\frac{\Delta m}{A_{m}\Delta t}$$

where, J is the mass flux in kg/(m^2^ h), Δm is the mass change in kg, Δt is the time interval in hours (h) and $A_{m}$ is the effective membrane area in m^2^.

The separation factor (SF) measures the membrane’s ability to reject one solvent from another. Within this work, SF is defined as the rejection of the ethanol compared to water. A SF of less than one indicates that the membrane was permeating more water than ethanol, whereas a SF of more than one indicates that the permeation of ethanol through the membrane was higher than that of water. A SF of one indicates that there was no separation between water and ethanol, and thus, the membrane was permeating both ethanol and water in the ratio of their feed concentration. The SF is given as follows:

(2) SF=wEPwwPwERwwR

where $w_{E}^{P}$ is the mass fraction of ethanol in the permeate, $w_{w}^{P}$ is the mass fraction of water in the permeate, $w_{E}^{R}$ is the mass fraction of ethanol in the retentate, and $w_{w}^{R}$ is the mass fraction of water in the retentate.

Two experiments were performed for each membrane listed in Table 2, with 100 wt% water as feed and 10 wt% ethanol solution (rest water) as feed, respectively. For each experiment, the feed tank was filled with 200 g of feed, and the tank was closed with clamps and pressurised slowly to 30 bars. The permeate was collected in a glass bottle. The experiment was terminated when 100 g of permeate had been collected. To calculate the SF (in the case of 10 wt% ethanol feed), permeate and retentate samples were collected and analysed via gas chromatography (GC) to measure the ethanol concentration (see Section 2.5.5).

#### 2.4.3. Determining the Number of Stages, the Amount of Water Required, and Particle Rejection during Discontinuous Diafiltration

The discontinuous diafiltration was first performed with a 5 wt% aqueous ethanol solution as feed to obtain the number of diafiltration stages and amount of water required to reduce the ethanol concentration below 0.5 wt% (ICH guidelines). Stage 0 of discontinuous diafiltration refers to pressurising the membrane module to reduce the initial feed mass by half by permeating the liquid through the membrane while retaining crystals and excipients. Thus, for stage 0, 200 g of feed was added to the feed tank, and 100 g of permeate was collected. Each subsequent stage (stages 1 to 3) corresponded to adding 100 g of water to the retentate (feed left after permeating 100 g of liquid) of the previous stage upon depressurising the membrane module and then pressurising again to reduce the feed mass to half. Thus, for stage 1, 100 g of water was added to the retentate from stage 0, and the process was repeated. This was continued until the concentration of ethanol in the permeate collected was below 0.5 wt% (ICH guidelines).

Similarly, discontinuous diafiltration was also performed with 0.1 wt% model particles, i.e., sipernat 500 LS particles in this case having a mean particle size of 6 µm (to simulate submicron particles) in a 5 wt% aqueous ethanol solution (solid-free basis) to evaluate particle rejection via dynamic light scattering (refer to Section 2.5.1).

#### 2.4.4. Evaluating Influence of Excipient on Permeate Flux

To evaluate the impact of excipient on the permeate flux, filtration was performed with (1) an aqueous 0.1 wt% HPMC E3 solution as feed and (2) an aqueous 0.1 wt% HPMC E3 and 0.1 wt% model particle suspension as feed. The filtration experiments were carried out using the experimental setup shown in Figure 1A. During filtration, 200 g of the feed was added to the membrane module, which was then pressurised using nitrogen gas to reach a pressure of 30 bars. The experiment was terminated once 100 g of permeate had been collected by depressurizing the membrane module.

#### 2.4.5. Solvent Exchange of Naproxen Crystal Suspension via Discontinuous Diafiltration

Solvent exchange of naproxen crystal suspensions was performed both via discontinuous diafiltration and semi-continuous diafiltration. Discontinuous diafiltration experiments were performed using the setup shown in Figure 1A in the same way as explained in Section 2.4.3. In addition, the feed and retentate samples were analysed for particle size distribution (PSD), polymorphic forms of crystals, ethanol, and HPMC E3 concentrations according to the procedures described in Section 2.5. The particle-size distribution of the retentate was also determined over time. After the solvent exchange, the retentate suspension was collected in a glass bottle and stored under stirred conditions for 6 days at 25 °C. The suspension was later stored without stirring at 25 °C for long-term stability analysis.

#### 2.4.6. Solvent Exchange of Naproxen Crystal Suspension via Semi-Continuous Diafiltration

The semi-continuous diafiltration setup shown in Figure 1B was used. First, 100 g of naproxen crystal suspension was added as feed, and then pressurisation was done. Once 50 g of permeate had been collected, the dilutant pump was started for the continuous addition of water while maintaining the membrane module at 30 bars. Permeate samples were collected at regular intervals. The process continued until the concentration of ethanol in the permeate was below 0.5 wt%. Feed, permeate, and retentate samples were analysed as mentioned below.

### 2.5. Analysis

#### 2.5.1. Particle Rejection

The rejection of model particles was investigated using dynamic light scattering (DLS) equipment, DynaPro Nanostar^®^, by Wyatt, CA, USA. It calculates the translational diffusion coefficient based on the temporally changing light intensity in solution (using an autocorrelation function).

#### 2.5.2. Particle Size Distribution (PSD)

To investigate the influence of solvent exchange on the API crystal properties and the stability of the API crystals in aqueous media, the PSD of crystals was investigated using laser diffractometry via the Mastersizer 3000 and HydroEV provided by Malvern Instruments (Malvern, UK). The analysis was performed on both feed and retentate samples. For the measurement, water was used as the dispersion medium, maintained under stirred conditions (2000 rpm) in a glass beaker. Samples from the suspension were added to the measuring beaker until the laser shading value was between 10% and 20%. Afterwards, the particle size was determined.

#### 2.5.3. A Polymorphic Form of Crystals

Samples of naproxen crystal suspensions before (feed) and after (retentate) the solvent exchange were air-dried at 60 °C for 24 h in a drying cabinet from Binder GmbH (Tuttlingen, Germany). The polymorphic form of dried solids was determined using a powder X-ray diffractometer (PXRD) Mini Flex 600 from Rigaku (Neu-Isenburg, Germany). The voltage and current were 40 kV and 15 mA, respectively. Data collection was performed in step-scan mode with a step size of 0.02° in the region of 2° ≤ 2θ ≤ 35°.

#### 2.5.4. Membrane Swelling Test

Membrane swelling indicates the ability of a membrane to absorb the solvent of interest. Swelling tests were performed with the membranes in 20 mL glass vials (VWR International, Dramstadt, Germany). The Duramem membrane had preservatives that needed to be washed out before the test, thus it was soaked in ethanol for 24 h prior to the test. After that, the membrane was cut into small pieces, and their mass was measured. at 25 °C, membrane pieces of known mass were then immersed in ethanol and another in water. After 24 h, the samples were withdrawn and weighed again. The membrane swelling was determined by the following equation:

(3) $$Swelling=\frac{m_{1}-m_{0}}{m_{0}}\times 100$$

where *swelling* is given in percentage, $m_{1}$ is the mass of membrane after swelling and $m_{0}$ is the mass of the membrane before swelling. This protocol was adapted from *Ho and Sirkar* [34].

#### 2.5.5. Concentration of Ethanol in Suspension

Concentration of ethanol in API crystal suspensions was determined by gas chromatography (GC) performed using a Shimadzu GC-14A equipped with FFAP-Innopeg column (CS-Chromatographie service, Langerwehe, Germany) and a flame ionization detector (FID). Hydrogen 5.0 and synthetical air 5.0 were used for the flame of the FID. Acetonitrile was the solvent, whereas helium 5.0 was used as the mobile phase. Samples were filtered through a 0.2 µm Polyethylene terephthalate (PET) syringe filter (Chromafil^®^ Xtra, Duren, Germany) prior to analysis.

#### 2.5.6. Concentration of HPMC E3 in Suspension

To investigate the retention of HPMC E3 by the membrane, the concentration of HPMC E3 was determined via an evaporative light scattering detector (ELSD) coupled with a high-performance liquid chromatography (HPLC) system from Agilent 1200 series (Ratingen, Germany). The feed, permeate, and retentate samples were analysed. The samples were filtered through 0.2 µm PET syringe filters prior to injection. The mobile phase was comprised of 40% acetonitrile and 60% water (on a volume basis) and flowed at a rate of 1 mL min^−1^. ELSD was maintained at 60 °C, 3.5 bar pressure, and a gain value of 4. Injection volume was 20 µL.

#### 2.5.7. Viscosity

The viscosity of the 0.1 wt% model particle suspension in water was measured using the Lovis 2000 M/ME rolling ball viscometer (Anton Paar, Graz, Austria) at 20 °C.

## 3. Results and Discussion

### 3.1. Membrane Selection for the Solvent Exchange Process

A suitable membrane for the solvent exchange has to offer (1) stability in the solvents of interest (within this work, water and ethanol), (2) a reasonably high flux (≥10 kg (m^2^ h)^−1^) through the membrane during the operations, and (3) a SF between the organic solvent and water equal to or higher than one to avoid excess usage of water during diafiltration. Five commercial OSN membranes (Table 2) were screened during membrane filtration based on their performance in terms of stability, permeate flux, and separation factor. Two feeds were used: (1) a mixture of 10 wt% ethanol and 90 wt% water representing the concentration of solvent/anti-solvent mixtures after the API anti-solvent crystallisation process (going into diafiltration) and (2) 100 wt% water as feed representing the retentate concentration at the end of the diafiltration process. It is important to note that, depending on the crystallisation process, the concentration of the solvent in the solvent/anti-solvent mixtures can be lower than 10 wt%. However, to investigate the performance of membranes with a reasonably high concentration of ethanol after the anti-solvent crystallisation, the feed with 10 wt% ethanol was used for membrane screening.

**Figure 2:**
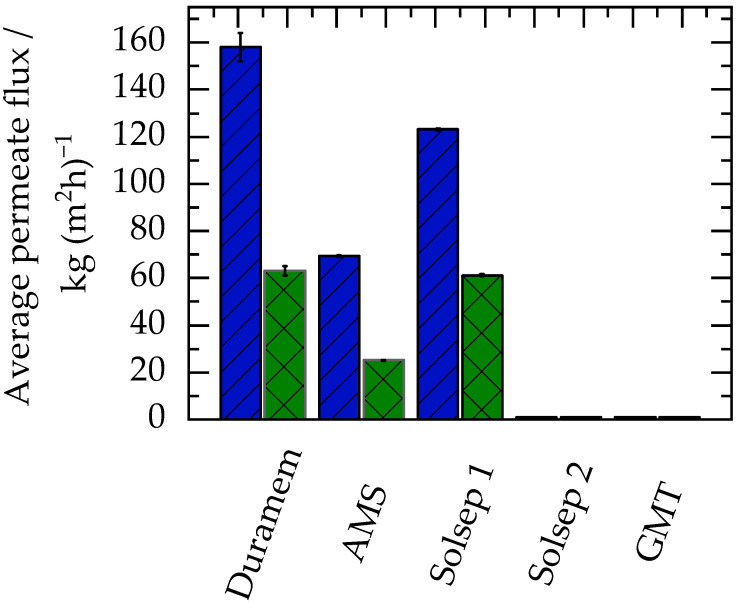
Average permeate flux for different OSN membranes at 30 bars and 25 °C. The x-axis indicates the names of the membranes used in this study. Details of the membranes are shown in Table 2. Blue bars with a line pattern indicate permeate flux for the diafiltration with a feed of 100 wt% water, whereas green bars with a cross pattern represent permeate flux for the diafiltration with 10 wt% ethanol in the aqueous feed.

The investigations showed that all membranes were stable in the ethanol/water mixtures; no dissolution or decoloration of the membranes was observed after the filtration experiments. Figure 2 shows the average permeate flux during the diafiltration experiments. Supplementary information includes the tabulated data of average permeate flux (Table S1) and permeate flux v/s time data (Table S2).

Solsep2 and GMT membranes are highly hydrophobic, as no wetting of the membrane surface was observed when removed from the filtration cell. This is the reason why no permeation of water or of the water/ethanol mixture through the Solsep2 and GMT membranes was observed.

Duramem, AMS, and Solsep 1 membranes are hydrophilic and, thus, showed permeate fluxes higher than 20 kg/(m^2^ h)^−1^ in all cases. It can also be seen from Figure 2 that in the case of 100 wt% water feed, the permeate flux was higher compared to the feed with 10 wt% ethanol and 90 wt% water. This was observed since all membranes had a higher affinity for water compared to ethanol. Moreover, the viscosity of water (0.890 mPa s at 25 °C [35]) is lower than that of water/ethanol mixtures (1.2606 mPa s at 25 °C for a 10 wt% ethanol solution [36]), aiding the transport through the membranes. A higher flux with increasing water content could be beneficial during diafiltration, where water is used as a dilutant. With each stage, as the water concentration increases, an increase in flux can be achieved, thereby reducing the overall time of filtration (and/or offering a lower membrane area requirement).

The SFs for the SolSep2 and GMT membranes could not be determined due to the lack of permeation through these membranes. Duramem and Solsep1 membranes showed a SF of one, which indicates that the membrane did not provide any separation between water and ethanol. As a result, the concentration of water and ethanol in the permeate was the same as in the feed. However, the AMS membrane had a low separation factor of 0.5, indicating much higher water permeation compared to ethanol. This could result in an increased water requirement during solvent exchange. Thus, in order to minimise wastewater and reduce water requirements, membranes with high SF should be preferred.

Results of filtration experiments for membrane screening show that Duramem performed better compared to other membranes in flux and SF and was thus selected for the next solvent exchange experiments.

### 3.2. Determining the Number of Stages and the Amount of Water Required for Solvent Exchange

Discontinuous diafiltration was performed with 5 wt% ethanol and 0.1 wt% model particles in an aqueous feed at 25 °C and 30 bar. The objective of conducting the diafiltration experiments with model particles was to mimic the retention of targeted crystals of size 6 μm (Dv50). Moreover, with these diafiltration experiments, the amount of water required to reduce the ethanol concentration below 0.5 wt% (as per ICH guidelines) was estimated without encountering the effect of other components of naproxen crystal suspension (e.g., excipients and dissolved API). The feed concentration of 5 wt% ethanol was chosen to keep residual solvent comparable to later experiments in which solvent exchange of naproxen crystal suspension was performed via diafiltration.

**Figure 3:**
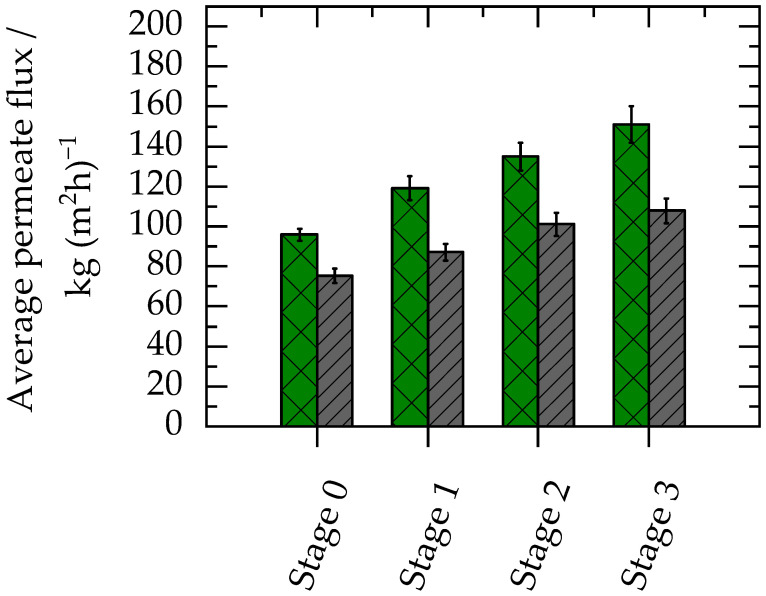
Average permeate flux during discontinuous diafiltration at different stages. During each stage, the initial feed mass is reduced by half, and an equivalent amount of water is added as feed for the next stage. The green crossed bars represent the results for a solution containing 5 wt% ethanol in water, and the grey lined bars show the flux for an aqueous feed containing 0.1 wt% model particles and 5 wt% ethanol (rest water). Experiments were carried out at 30 bar and 25 °C.

Figure 3 represents the average permeate flux during each stage of discontinuous diafiltration. The permeate flux profile with respect to time has been tabulated in the supporting documents (Tables S3 and S4).

To reach the desired ethanol concentration in the permeate, the process was completed in four stages. Table 4 presents the feed concentrations of ethanol and model particles during different stages of the discontinuous diafiltration.

During the first stage (stage 0), the initial overall feed mass was reduced to half, thereby increasing the concentration of crystals. This step was performed to reduce the overall water requirement of the process. For all the subsequent stages (stages 1, 2, and 3), the same amount of water (as permeate collected in the previous stage) was added to the feed to have the same starting concentration of crystals for each stage. The process was completed with a water consumption of 1.5 g of water per g of feed added. Permeate flux was constant over time during filtration, and thus an average value of flux is reported for each stage in Figure 3.

During the diafiltration, the permeate flux is the most important performance parameter of the membrane process, as this can provide useful information about filtration time and membrane area requirements for industrial-scale operations. Higher flux can result in a smaller membrane area requirement, thus reducing the overall cost of the process. During filtration, it was observed that the permeate flux increased from stage to stage of solvent exchange. The increased flux at later stages was a result of the decreasing ethanol concentration and increasing water concentration, which resulted in a decrease in the viscosity of the feed with each stage. In addition, the Duramem membrane had a higher affinity for water compared to ethanol. The higher affinity was confirmed by swelling tests, which indicated that the membrane swelled by 25% with water but only by 6% with ethanol (determined by measuring the change in mass; see 2.5.4. Membrane Swelling Test). It is important to note that the swelling had no negative impact on solute retention in this case as the molecular weight cut-off of the membrane was low enough (i.e., 300 Da, corresponding to a pore size of less than 1 nm) compared to the targeted particle size (i.e., ≥0.1 µm). DLS measurements of the permeate samples indicated that no particles were present in the permeate. Following this, complete particle retention was achieved for all concentration stages.

The use of model particles can give an efficient first insight into the permeate flux behaviour during filtration with API crystal suspension by mimicking the presence of crystals in the feed. With addition of the model particles, a 30% to 40% reduction in flux was observed compared to the filtration with 5 wt% ethanol in aqueous feed as shown in Figure 3. The reduction of flux with the addition of the model particles was due to the increase in viscosity caused by the addition of the model particles. The viscosity of water is 0.890 mPa s at 25 °C [35], whereas with the addition of 0.1 wt% model particles in water, the viscosity increased to 1.343 mPa s. In addition, the decrease in the flux resulted from the formation of a layer of particles on the membrane surface, which was observed after the experiments. This phenomenon is common in membrane filtration, particularly in dead-end mode when the flow direction is perpendicular to the membrane and the particles in the feed are pushed towards the surface of the membrane [37]. From these experiments, we conclude that the solvent exchange of a naproxen suspension with 5 wt% ethanol can be completed in a four-stage diafiltration process with a water requirement of 1.5 g of water per g of feed added.

### 3.3. Investigating the Influence of Excipients on Membrane Performance during Solvent Exchange

**Figure 4:**
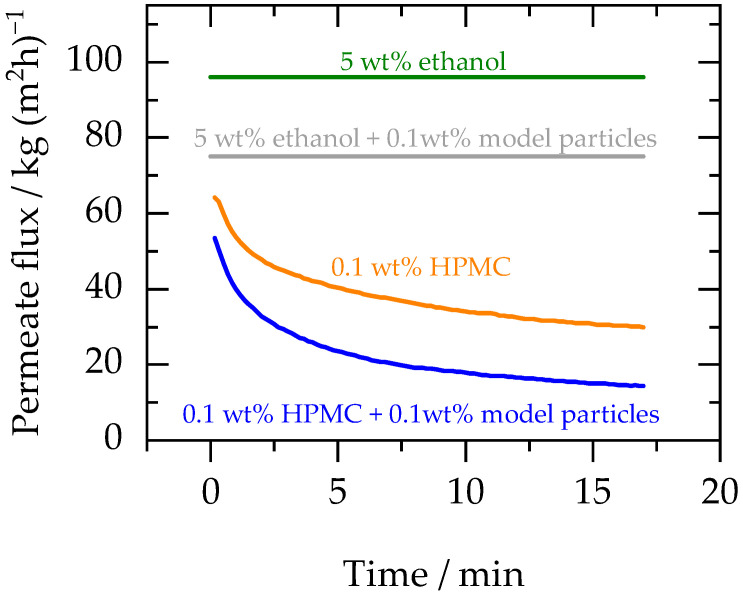
Green line indicates the permeate flux profile for the diafiltration with an aqueous feed containing a 5 wt% ethanol solution. The grey line indicates the permeate flux profile of an aqueous feed containing 0.1 wt% model particles and 5 wt% ethanol. Orange line represents the permeate flux profile for the feed with 0.1 wt% HPMC E3 solution (in water), and blue line shows the permeate flux profile for the feed consisting of 0.1 wt% model particles and 0.1 wt% HPMC E3 in water. Experiments were performed at 30 bar and 25 °C.

To understand the influence of excipients (that are present during diafiltration) on the membrane performance, diafiltration was performed with an aqueous feed containing: (1) 0.1 wt% HPMC E3 solution (polymeric excipient stabilising naproxen crystals) and (2) 0.1 wt% HPMC E3 plus 0.1 wt% model particles. Figure 4 shows the permeate flux profiles obtained during these experiments along with the permeate flux profile obtained with aqueous feed containing 5 wt% ethanol and aqueous feed with 0.1 wt% model particles plus 5 wt% ethanol (tabulated data is provided in supporting information Table S5).

As shown in Figure 4, the highest flux was achieved for the filtration performed with 5 wt% ethanol in the aqueous feed (neither model particles nor HPMC E3). Flux dropped with the addition of model particles due to higher viscosity and the accumulation of particles on the membrane surface (as explained in Section 3.2). However, HPMC E3 had the most significant influence on the permeate flux as it (1) reduced the flux compared to the feed solution containing model particles and 5 wt% ethanol in water and (2) changed the flux profile from a straight line to a decreasing curve. The initial fast drop in flux was contributed by an inherit phenomenon called concentration polarization. During this, the concentration of the solute near the membrane surface increases rapidly compared to the bulk, resulting in a fast decline of the flux during the first couple of minutes of filtration [37]. This phenomenon was more dominant for solutions containing HPMC E3 (where an initial flux drop was observed) as compared to those containing only suspended model particles (where no initial flux drop was observed), because it occurs only for molecules that remain dissolved in the feed (HPMC E3 in this case) [38]. During industrial applications, it can be controlled or reduced with more rigorous mixing close to the membrane. To improve mixing during membrane filtration, a crossflow membrane setup can be used (instead of a dead-end setup), where mixing can be controlled more efficiently by changing the crossflow velocity.

From these results, it can be concluded that the polymeric excipients (e.g., HPMC E3) used for stabilisation of crystals in suspensions have a significant influence on the membrane performance. If the selected excipient used during crystallisation adversely affects the membrane’s performance in terms of flux, alternate excipient systems can be explored at an early stage of formulation development. However, if a certain excipient is crucial for the formulation, different membrane materials, membrane modules, and operating modes can be tested to improve the overall flux, which is beneficial in terms of reducing the membrane area requirement.

### 3.4. Case Study: Removing Ethanol from a Naproxen Crystal Suspension after Anti-Solvent Crystallisation

**Figure 5:**
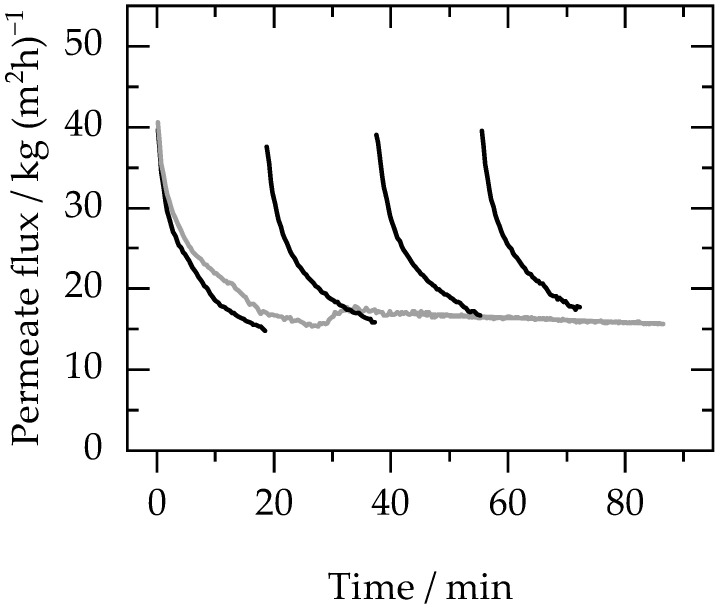
Permeate flux profile over time during discontinuous (black lines) and semi-continuous (grey lines) diafiltration of naproxen crystals (0.2 wt%) suspended in a solution of 0.1 wt% HPMC E3, 3.8 wt% ethanol, and 95.9 wt% water as mentioned in Table 3. The suspension used in both cases has the same composition. Experiments were conducted at 30 bar and 25 °C.

The solvent exchange of naproxen crystal suspension (the feed composition is mentioned in Table 3) was carried out via a discontinuous diafiltration process (refer to Section 2.4.5) as well as a semi-continuous diafiltration process (refer to Section 2.4.6). Figure 5 shows the permeate flux profiles for the two diafiltration modes (tabulated data is available in Table S6 of the supporting information).

The experimental results show that in the case of discontinuous diafiltration, the flux dropped as the feed was reduced to half and then the membrane module was depressurized to add water for the next stage. On repressurization, initial flux was restored. In contrast, in the case of semi-continuous diafiltration, the flux dropped very rapidly as the feed mass was being reduced to half (as explained in Section 2.4.6), and once the flow of water was started (at around a 20-min time interval), the flux stabilized. The input flow rate of water was kept the same as the permeate flow rate to maintain constant concentrations of particles and excipients inside the membrane module.

The filtration time for discontinuous diafiltration (72 min) was less than for semi-continuous diafiltration (86.5 min). This is because the average flux in discontinuous diafiltration was higher (22 kg (m^2^ h)^−1^) as compared to semi-continuous diafiltration, where the flux was lower (the average flux is 18 kg (m^2^ h)^−1^). The average flux in the case of discontinuous diafiltration was higher because the starting flux of each stage during the discontinuous was similar, indicating that depressurization of the membrane module restored the flux by removing the adsorbed particles on the membrane surface and that there was no permanent fouling on the membrane. However, in case of semi-continuous diafiltration, the flux did not restore after the initial drop in flux during the preconcentration stage (i.e., until the initial feed mass reduces to half). This was because of the particle adsorption on the surface of the membrane.

During discontinuous diafiltration, the ethanol concentration dropped after each stage as the water was added, as shown in Figure 5. Table 5 shows the water added during each stage and corresponding ethanol concentration.

After four stages, the ethanol concentration was reduced from 3.85 wt% to 0.4 wt%, which was below the residual solvent limit permitted according to the ICH guidelines. The amount of water required during the discontinuous process was 1.5 g of water per g of feed (this corresponds to 750 kg of water per kg of API-purified), which is comparable to the results obtained with the solution containing model particles (see Section 3.2). During semi-continuous diafiltration, water was continuously added until the ethanol concentration in the permeate was below 0.5 wt%. In this case, the amount of water required was 1.3 g of water per g of feed (this corresponds to 650 kg of water per kg of API-purified). It is important to note here that the objective of this study is to provide a proof of concept for a solvent exchange process and understand its impact on the stability of crystals in long-acting suspensions. Higher freshwater consumption or the generation of wastewater can be a point of concern. Therefore, future studies are needed to optimise the process so that the water requirements can be minimized. Moreover, a recycling strategy can be developed to minimise the solvent-based wastewater. It is particularly important to consider wastewater minimization and treatment for toxic or non-biodegradable solvents.

The discontinuous diafiltration mode, even though it offers an advantage in terms of flux, is not preferred at industrial scales where large quantities need to be treated in a continuous mode. Therefore, a compromise needs to be found in terms of flux to take advantage of things like continuous processing, handling of larger quantities, and minimization of process downtime by adopting continuous diafiltration. However, for applications where small batches need to be treated, discontinuous diafiltration can be used to improve membrane performance in terms of flux.

**Figure 6:**
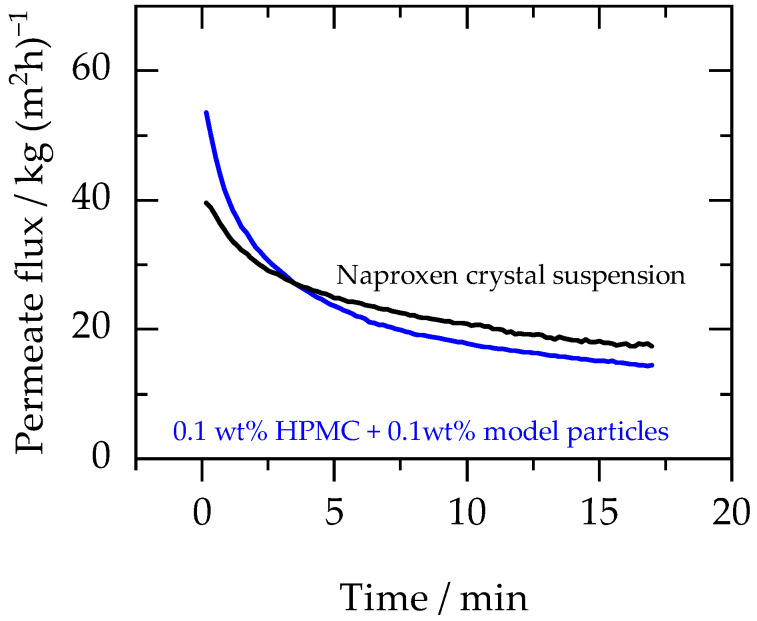
Permeate flux profile of naproxen crystal suspension (initial crystal load: 0.2 wt% and HPMC E3 concentration: 0.1 wt%) (black) and aqueous suspension of 0.1 wt% model particles plus 0.1 wt% HPMC E3 (blue). Experiments were performed at 30 bars and 25 °C.

To verify the suitability of the use of model particles in process design, the permeate flux profile obtained during the discontinuous diafiltration of the naproxen crystal suspension (which contained 0.2 wt% naproxen crystals and 0.1 wt% HPMC E3 in water) was compared with the permeate flux profile obtained during the filtration of the suspension containing 0.1 wt% model particles and 0.1 wt% HPMC E3 in water. The profiles are shown in Figure 6.

The experimental results indicate that the profiles were similar in terms of flux behaviour i.e., rapid initial drop followed by a steadier decline. The nature of the particles was different in both cases; however, it did not significantly influence the flux profile. Thus, the suspension with model particles and the polymeric excipient used to stabilize the API crystals can be used to predict the membrane performance for various API crystal/excipient systems during the membrane-based solvent exchange process. 

In this study, a very small membrane cut-off was selected to avoid any disturbance in the stability of the crystals due to excipient loss. However, for industrial applications a membrane with higher cut-off (cut-off smaller than the size of crystal but bigger than the size of the excipient) can be used for solvent exchange if the stability of crystals can be preserved with the loss of the excipients or if the excipients are bounded to crystals and are retained by the membrane. Therefore, for the evaluation of the membrane-based diafiltration process, it is crucial that the stability of the API crystals is monitored after the solvent exchange process.

**Figure 7:**
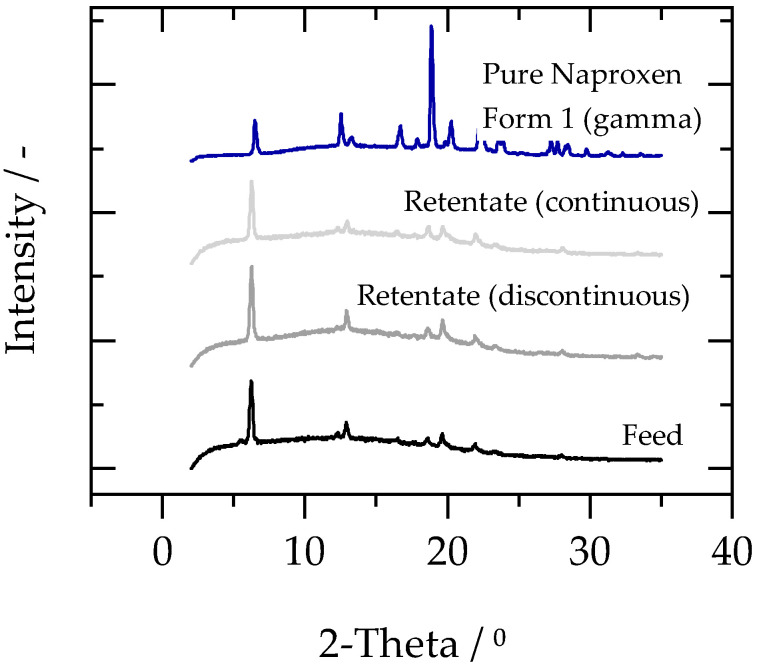
X-ray powder diffraction patterns of pure naproxen powder form 1 (blue), naproxen in dried feed (black), retentate of discontinuous diafiltration (grey) and retentate of semi-continuous diafiltration (light grey).

Figure 7 shows the X-ray diffraction patterns of the feed and retentate (dried) naproxen crystals in the cases of both semi-continuous and discontinuous diafiltration (the feed in both cases was the same and produced in one batch).

All different X-ray powder diffraction patterns are very similar. The characteristic peaks at 6.2°, 12.9°, 18.5°, and 19.6° are in the same position for all samples and are in good agreement with the characteristic peaks of crystal form 1 (gamma) of naproxen (blue line), which is considered to be the most stable polymorphic form [39]. However, the intensity of the middle peaks was suppressed when compared to pure naproxen. This could arise from the presence of HPMC E3 along with the naproxen in the dried feed and retentate samples. Nevertheless, similar X-ray diffraction patterns in feed and retentate samples indicate that the solvent exchange process had no impact on the polymorphic form of the crystals. This is beneficial because a change in the polymorphic form of the crystals can impact the pharmacokinetics and dissolution properties of the API. Therefore, it is important for the process design that the crystals’ polymorphic form is preserved.

Table 6 shows the particle size distribution (volume-based) measured via laser diffraction of naproxen crystals in the suspension before (feed) and after (retentate) the solvent exchange via both discontinuous and semi-continuous diafiltration.

The experimental results show that the particle sizes of naproxen crystals in feed and retentate were similar. Moreover, it also remained stable for up to 3 months (93 days) after solvent exchange. The excipient used for stabilisation during crystallisation, i.e., HPMC E3, was effective and helped in maintaining good stability of the naproxen crystals in aqueous media. However, slight agglomeration was observed in the case of semi-continuous diafiltration (indicated by a higher Dv90). This was because during the semi-continuous diafiltration, the continuous pressurisation resulted in cake formation on the membrane surface and might have caused crystal agglomeration. This increase in Dv90 was also observed when the retentate from discontinuous diafiltration was stored without stirring and the settling of particles was observed (as was also observed in the case of the feed sample). For further investigations, it is suggested to use some resuspending agents that can minimise the settling. However, the agglomeration was not caused by solvent exchange, as it was also observed in the feed samples stored under similar conditions. Thus, we concluded that the stability of crystals in suspension remained intact after the solvent exchange process in terms of PSD and polymorphic form.

## 4. Conclusions

In this work, a membrane-based diafiltration process has been applied for the purification of naproxen crystal suspensions. The process successfully reduced the concentration of residual organic solvent (ethanol) as per ICH guidelines (i.e., ≤0.5 wt%) while keeping the stability of crystals in suspension intact (polymorphic form and particle size remain stable after the solvent exchange). This study reveals that membranes for industrial applications can be selected based on their stability in the solvent/anti-solvent system used and on the targeted crystal size distribution (ultrafiltration membranes can be used in cases of larger crystal sizes). We suggest using a model system (i.e., a suspension of model particles and excipient) as a predictive tool to effectively select the right membrane size and material. In this way, the experimental burden during the implementation of the solvent exchange process on a new system can be reduced significantly. Our novel approach is scalable, less energy-intensive, and provides better control over crystal properties. Following that, this work provides the proof of concept for establishing a process for organic solvent removal and bringing the production of API crystal suspension by using solvent/anti-solvent crystallisation one step closer to commercialization. The experimental framework suggested in this work can serve as the foundation for the design of experiments implementing solvent exchange in various API systems. For scale-up studies, it is suggested to validate the process using a crossflow membrane setup, which can reduce concentration polarisation and fouling as well as allow processing higher volumes.

## Figures and Tables

**Table 1:** List of materials used in this study with chemical abstract service (CAS) number, purity, and supplier information.

Material	CAS	Purity/%	Supplier
Naproxen	22204-53-1	98	Sigma Aldrich, St. Louis, MO, USA
Hydroxypropyl methylcellulose E3 (HPMC E3)	9004-65-3	99	Dow Wolf Cellulosics Gmbh, Bomlitz, Germany
Ethanol	64-17-5	99.9	Merck KGaA, Dramstadt, Germany
Acetonitrile	75-05-8	99.9	Carl Roth Gmbh, Karlsruhe, Germany
Model particles (Sipernat 500 LS)	112926-00-8	98.5	Evonik Industries AG, Wesseling, Germany
Deionized water	-	-	MilliQ^®^, Merck KgaA, Dramstadt, Germany
Hydrogen 5.0	1333-74-0	99.99	Messer Industriegase GmbH, Bad Soden, Germany
Synthetical air 5.0	-	99.99	
Helium 5.0	7440-59-7	99.99	
Nitrogen (techn.)	7727-37-9	99.9	

**Table 2:** List of OSN membranes used within this work with supplier, membrane material, molecular weight cut-off (MWCO), maximum operating pressure, and maximum operating temperature.

Label Used in the Paper	Product Name	Supplier	Membrane Material	Molecular Weight Cut-Off (MWCO)/Da	Maximum Pressure/Bar	Maximum Temperature/°C
Duramem	DuraMem 300	Evonik, Marl, Germany	Polyimide	300 ^a^	60	50
AMS	AMS NanoPro	AMS Technologies, Or Yehuda, Israel	Not specified	- ^b^	70	60
Solsep1	SolSep 090101	SolSep BV, Apeldoorn, Netherlands	Not specified	350 ^a^	20	80
Solsep2	SolSep 070706			250 ^a^	20	100
GMT	GMT_oNF	Borsig Membrane Technology, Gladbeck, Germany	Silicon polymer-based composite	- ^c^	15–30	60

^a^: based on supplier’s information. ^b^: showed glucose rejection of > 96% as per supplier. ^c^: showed hexatriacontane (MW 507 g mol^−¹^) rejection of 98% and tetracosane (MW 339 g mol^−¹^) rejection of 75% as per the supplier’s information.

**Table 3:** Operational parameters for the production of naproxen crystal suspension via solvent/anti-solvent crystallisation and the final composition of the suspension.

Parameters	
Ethanol side flow rate/mL min^−1^	12
Water side flow rate/mL min^−1^	112
Residence time in y-connector/s	0.1
Temperature/°C	25
Concentrations in crystal suspension ^a^/wt%	
Naproxen	0.2
HPMC E3	0.1
Ethanol	4.0
Water	95.7

^a^: based on mass balance.

**Table 4:** Feed concentrations during different stages of discontinuous diafiltration.

Feed Concentrations	Stage 0	Stage 1	Stage 2	Stage 3
Ethanol ^a^/wt%	5	2	1	0.1
Model Particles ^b^/wt%	0.1	0.1	0.1	0.1
Ethanol ^b^/wt%	5	2	1	0.4

^a^: Diafiltration with a 5 wt% ethanol solution. ^b^: Diafiltration with an aqueous feed containing 0.1 wt% model particles and 5 wt% ethanol.

**Table 5:** Amount of water added and ethanol concentration during different stages of discontinuous diafiltration of naproxen crystal suspension.

	Feed	Stage 0 Permeate	Stage 1 Permeate	Stage 2 Permeate	Stage 3 Permeate	Retentate
Feed or water added/g	90	-	45	45	45	-
Ethanol concentration/wt%	3.85	3.09	2.12	1.10	0.57	0.41

**Table 6:** Particle size distribution (volume-based) of naproxen crystals in suspension before (feed) and after (retentate) the solvent exchange.

	Particle Size Distribution (PSD)							
Distribution	Unit	Feed	Retentate (Discontinuous)			Retentate (Semi-Continuous)		
			Day 0	Day 6 ^a^	Day 93 ^b^	Day 0	Day 6 ^a^	Day 93 ^b^
Dv10	μm	2.4 ± 0.1	2.4 ± 0.1	2.1 ± 0.1	1.8 ± 0.1	2.3 ± 0.1	2.1 ± 0.1	2.1 ± 0.1
Dv50	μm	7.7 ± 0.1	7.6 ± 0.1	6.2 ± 0.1	6.5 ± 0.1	8 ± 0.1	6.7 ± 0.1	6.8 ± 0.1
Dv90	μm	31 ± 2	30 ± 2	32 ± 2	43 ± 2	42 ± 2	41 ± 2	43 ± 2

^a^: Stored at 25 °C in a shaker with 130 rpm. ^b^: Stored at 25 °C without stirring/shaking.

## Data Availability

Data is contained within the article.

## Supplementary Materials

The following supporting information can be downloaded at: https://www.mdpi.com/article/10.3390/membranes13030263/s1. Table S1. Average permeate flux of different OSN membranes at 30 bar and 25 °C. Tabulated data from Figure 2 of manuscript. Table S2. Permeate flux v/s time data of different OSN membranes at 30 bar and 25 °C. Table S3. Permeate flux v/s time data from diafiltration experiment with feed containing 5 wt% ethanol (rest water). Corresponds to the Figure 3 of the manuscript. Table S4. Permeate flux v/s time data for diafiltration with an aqueous feed containing 0.1 wt% model particles and 5 wt% ethanol (rest water). Corresponds to the Figure 3 of the manuscript. Table S5. Permeate flux profile for the diafiltration with an aqueous feed containing: (1) 0.1 wt% HPMC E3 solution (in water), (2) 0.1 wt% model particles and 0.1 wt% HPMC E3 in water, (3) 5 wt% ethanol solution and (4) an aqueous feed containing 0.1 wt% model particles and 5 wt% ethanol. Corresponds to Figure 4 in the manuscript. Table S6. Permeate flux profile over time during discontinuous and semi-continuous diafiltration of naproxen crystals (0.2 wt%) suspended in a solution of 0.1 wt% HPMC E3, 3.8 wt% ethanol and 95.9 wt% water. The suspension used in both cases had the same composition. The data corresponds to the Figure 5 of the manuscript.

## Abbreviations

Abbreviation	Description
API	Active pharmaceutical ingredient
DLS	Dynamic light scattering
ELSD	Evaporative light scattering detector
FEP	Fluorinated ethylene propylene
FID	Flame ionisation detector
GC	Gas chromatography
HPLC	High-performance liquid chromatography
HPMC E3	Hydroxypropyl methylcellulose E3
ICH	International Council for Harmonisation
MW	Molecular weight
MWCO	Molecular weight cut-off
OSN	Organic solvent nanofiltration
PET	Polyethylene terephthalate
PSD	Particle size distribution
PTFE	Polytetrafluoroethylene
PXRD	Powder X-ray diffractometer
rpm	Revolution per minute
Symbols	Description
$A_{m}$	Effective membrane area
Δt	Time interval
Δm	Change in mass
$w_{E}^{P}$	Mass fraction of ethanol in the permeate
$w_{E}^{R}$	Mass fraction of ethanol in retentate
$w_{w}^{P}$	Mass fraction of water in permeate
$w_{w}^{R}$	Mass fraction of water in retentate
Dv10	Volume-based diameter of 10% of the sample
Dv50	Volume-based diameter of 50% of the sample
Dv90	Volume-based diameter of 90% of the sample
