# Supplementary material for: Membrane-Based Solvent Exchange Process for Purification of API Crystal Suspensions
Source: Membranes (Basel). 2023 Feb 23;13(3):263. doi: 10.3390/membranes13030263 (PMC10058991; doi:10.3390/membranes13030263)
Supplement: Supplementary file 1 [file membranes-13-00263-s001.zip › membranes-2218127-supplementary.pdf]

Membrane-based solvent exchange process for purification of API crystal suspensions

Fatima Anjum, Maximilian Wessner and Gabriele Sadowski*

Department of Biochemical and Chemical Engineering, Laboratory of Thermodynamics, TU Dortmund University, Emil-Figge-Str. 70, D-44227 Dortmund, Germany

***Correspondence:** Gabriele.sadowski@tu-dortmund.de (G.S.); Tel: +49-231-755-2635

Supporting Information

3.1. Membrane selection for the solvent exchange process

Table S1 Average permeate flux of different OSN membranes at 30 bar and 25 C. Tabulated data from figure 2 of manuscript.

Membranes	Feed Composition	Average Flux / kg (m² h)⁻¹	Error
**Duramem**	Pure Water	158	6
	10 wt% Ethanol	63	2
**AMS**	Pure Water	69.3	0.3
	10 wt% Ethanol	25.2	0.2
**Solsep 1**	Pure Water	123.1	0.5
	10 wt% Ethanol	61.2	0.5
**Solsep 2**	Pure Water	-	-
	10 wt% Ethanol	-	-
**GMT**	Pure Water	-	-
	10 wt% Ethanol	-	-

Table S2 Permeate flux v/s time data of different OSN membranes at 30 bar and 25 C.

AMS Pure Water		AMS 10 wt% Ethanol		Solsep 1 Pure Water		Solsep 1 10 wt% Ethanol		Duramem Pure Water		Duramem 10 wt% Ethanol	
**Time**	**Flux**	**Time**	**Flux**	**Time**	**Flux**	**Time**	**Flux**	**Time**	**Flux**	**Time**	**Flux**
**s**	**kg (m² h)⁻¹**	**s**	**kg (m² h)⁻¹**	**s**	**kg (m² h)⁻¹**	**s**	**kg (m² h)⁻¹**	**s**	**kg (m² h)⁻¹**	**s**	**kg (m² h)⁻¹**
0.0		0.0	32.2	10.0	128.1	10.0	69.3	7.0	132.4	7.0	7.6
10.0	73.3	10.0	29.3	20.0	137.1	20.0	68.0	14.0	141.0	14.0	11.4
20.0	72.0	20.0	30.3	30.0	132.9	30.0	69.3	21.0	132.4	21.0	10.5
30.0	67.3	30.0	27.3	40.0	130.9	40.0	64.7	28.0	138.1	28.0	18.1
40.0	76.0	40.0	29.4	50.0	129.0	50.0	64.6	35.0	142.9	35.0	23.8
50.0	71.3	50.0	27.5	60.0	126.8	60.0	62.0	42.0	136.2	42.0	21.9
60.0	71.3	60.0	26.6	70.0	130.9	70.0	63.3	49.0	138.1	49.0	23.8
70.0	73.3	70.0	26.8	80.0	124.2	80.0	66.0	56.0	143.8	56.0	27.6
80.0	73.3	80.0	26.7	90.0	130.7	90.0	72.7	63.0	136.2	63.0	26.7
90.0	71.9	90.0	25.7	100.0	125.0	100.0	70.0	70.0	153.3	70.0	28.6
100.0	71.3	100.0	26.7	110.0	127.8	110.0	72.6	77.0	130.5	77.0	28.6
110.0	71.3	110.0	25.6	120.0	124.1	120.0	69.3	84.0	136.2	84.0	32.4

120.0	73.3	120.0	25.5	130.0	126.7	130.0	68.0	91.0	139.0	91.0	36.2
130.0	72.6	130.0	23.6	140.0	124.0	140.0	68.0	98.0	129.5	98.0	41.0
140.0	71.3	140.0	27.4	150.0	122.3	150.0	66.0	105.0	135.2	105.0	42.9
150.0	72.6	150.0	25.5	160.0	125.0	160.0	66.0	112.0	137.1	112.0	41.0
160.0	73.3	160.0	25.7	170.0	121.6	170.0	66.6	119.0	133.3	119.0	42.9
170.0	72.6	170.0	26.0	180.0	125.2	180.0	61.3	126.0	134.3	126.0	43.8
180.0	70.6	180.0	23.9	190.0	122.9	190.0	64.0	133.0	140.0	133.0	45.7
190.0	70.6	190.0	26.8	200.0	124.6	200.0	64.0	140.0	129.5	140.0	57.1
200.0	71.9	200.0	26.4	210.0	124.6	210.0	61.3	147.0	133.3	147.0	54.3
210.0	72.0	210.0	23.2	220.0	124.0	220.0	63.3	154.0	133.3	154.0	48.6
220.0	73.9	220.0	25.7	230.0	120.9	230.0	56.7	161.0	129.5	161.0	51.4
230.0	70.6	230.0	25.6	240.0	126.4	240.0	59.3	168.0	149.5	168.0	58.1
240.0	70.6	240.0	24.1	250.0	120.0	250.0	62.0	175.0	125.7	175.0	50.5
250.0	70.6	250.0	24.7	260.0	127.6	260.0	62.7	182.0	133.3	182.0	57.1
260.0	72.7	260.0	23.6	270.0	119.6	270.0	60.7	189.0	139.0	189.0	70.5
270.0	70.0	270.0	26.4	280.0	122.3	280.0	67.3	196.0	126.7	196.0	58.1
280.0	74.0	280.0	25.7	290.0	124.0	290.0	66.7	203.0	135.2	203.0	63.8
290.0	70.6	290.0	24.5	300.0	122.5	300.0	66.7	210.0	137.1	210.0	72.4
300.0	70.7	300.0	23.7	310.0	121.3	310.0	61.3	217.0	128.6	217.0	62.9
310.0	70.0	310.0	25.1	320.0	120.7	320.0	66.0	224.0	136.2	224.0	76.2
320.0	71.3	320.0	25.9	330.0	125.3	330.0	63.3	231.0	142.9	231.0	70.5
330.0	71.3	330.0	23.1	340.0	120.6	340.0	64.7	238.0	131.4	238.0	67.6
340.0	72.0	340.0	24.8	350.0	123.2	350.0	63.3	245.0	136.2	245.0	74.3
350.0	69.3	350.0	24.7	360.0	119.4	360.0	62.0	252.0	136.2	252.0	64.8
360.0	69.3	360.0	24.2	370.0	120.3	370.0	63.3	259.0	132.4	259.0	72.4
370.0	71.3	370.0	25.0	380.0	124.3	380.0	63.3	266.0	152.4	266.0	71.4
380.0	70.0	380.0	25.7	390.0	121.4	390.0	64.0	273.0	128.6	273.0	64.8
390.0	69.3	390.0	23.8	400.0	122.7	400.0	61.3	280.0	134.3	280.0	73.3
400.0	69.3	400.0	24.8	410.0	114.2	410.0	63.3	287.0	139.0	287.0	61.9
410.0	70.0	410.0	25.2	420.0	123.4	420.0	63.3	294.0	166.7	294.0	68.6
420.0	72.0	420.0	24.2	430.0	122.3	430.0	66.0	301.0	140.0	301.0	68.6
430.0	68.0	430.0	24.6	440.0	119.5	440.0	56.7	308.0	138.1	308.0	68.6
440.0	71.3	440.0	25.8	450.0	121.5	450.0	63.3	315.0	381.0	315.0	66.7
450.0	70.0	450.0	23.7	460.0	121.4	460.0	62.0	322.0	131.4	322.0	66.7
460.0	67.3	460.0	25.8	470.0	120.8	470.0	65.3	329.0	149.5	329.0	63.8
470.0	70.0	470.0	22.6	480.0	119.9	480.0	60.7	336.0	379.0	336.0	68.6
480.0	71.3	480.0	24.6	490.0	122.4	490.0	62.0	343.0	129.5	343.0	68.6
490.0	69.3	490.0	26.0			500.0	60.6	350.0	133.3	350.0	60.0
500.0	70.0	500.0	25.4			510.0	65.3	357.0	379.0	357.0	67.6
510.0	69.3	510.0	23.6			520.0	61.8	364.0	139.0	364.0	66.7
520.0	68.6	520.0	23.8			530.0	56.6	371.0	127.6	371.0	71.4
530.0	68.0	530.0	25.4			540.0	63.3	378.0	352.4	378.0	42.9
540.0	71.3	540.0	23.9			550.0	58.6			385.0	60.0
550.0	67.9	550.0	26.1			560.0	57.3			392.0	67.6
560.0	67.3	560.0	24.5			570.0	64.0			399.0	65.7
570.0	70.6	570.0	26.0			580.0	57.3			406.0	64.8
580.0	69.3	580.0	24.2			590.0	60.0			413.0	61.0
590.0	66.6	590.0	23.9			600.0	59.3			420.0	61.9
600.0	70.0	600.0	23.7			610.0	58.0			427.0	65.7

610.0	68.0	610.0	27.7	620.0	59.3	434.0	64.8
620.0	67.9	620.0	22.7	630.0	60.6	441.0	71.0
630.0	70.6	630.0	24.2	640.0	60.0	448.0	61.9
640.0	68.6	640.0	23.9	650.0	54.0	455.0	49.5
650.0	66.0	650.0	24.2	660.0	64.6	462.0	139.0
660.0	68.6	660.0	25.5	670.0	58.6	469.0	63.8
670.0	68.0	670.0	24.6	680.0	58.6	476.0	71.4
680.0	68.6	680.0	24.2	690.0	57.3	483.0	113.3
690.0	68.6	690.0	24.6	700.0	58.6	490.0	68.6
700.0	68.0	700.0	24.6	710.0	62.0	497.0	56.2
710.0	66.7	710.0	25.5	720.0	58.0	504.0	135.2
720.0	66.7	720.0	23.1	730.0	58.6	511.0	61.9
730.0	69.3	730.0	22.0	740.0	58.0	518.0	52.4
740.0	65.3	740.0	22.6	750.0	60.0	525.0	135.2
750.0	67.9			760.0	60.6	532.0	49.5
760.0	68.0			770.0	54.6	539.0	63.8
770.0	67.3			780.0	58.0	546.0	135.2
780.0	68.7			790.0	63.3	553.0	61.0
790.0	66.6			800.0	56.6	560.0	63.8
800.0	66.6			810.0	58.6	567.0	136.2
810.0	66.0			820.0	58.0	574.0	61.0
820.0	68.0			830.0	58.0	581.0	61.9
830.0	66.6			840.0	57.3	588.0	141.9
840.0	67.3			850.0	57.3	595.0	51.4
850.0	65.9			860.0	59.3	602.0	59.0
860.0	65.3			870.0	60.0	609.0	130.5
870.0	65.3			880.0	58.6	616.0	61.0
880.0	67.3			890.0	58.0	623.0	63.8
890.0	67.3			900.0	56.6	630.0	117.1
900.0	65.3			910.0	58.0	637.0	53.3
910.0	66.6			920.0	61.3	644.0	65.7
920.0	65.3			930.0	55.3	651.0	121.0
930.0	66.7			940.0	57.3	658.0	56.2
940.0	66.6			950.0	58.6	665.0	52.4
950.0	65.3					672.0	121.9
960.0	66.0					679.0	60.0
970.0	64.6					686.0	57.1
980.0	66.0					693.0	107.6
990.0	64.0					700.0	61.9
						707.0	53.3
						714.0	117.1
						721.0	54.3
						728.0	54.3
						735.0	116.2
						742.0	43.8
						749.0	59.0
						756.0	108.6
						763.0	59.0
						770.0	48.6

777.0	111.4
784.0	41.9
791.0	53.3
798.0	103.8
805.0	53.3
812.0	54.3
819.0	101.0
826.0	45.7
833.0	55.2
840.0	102.9
847.0	51.4
854.0	49.5
861.0	96.2
868.0	57.1
875.0	52.4
882.0	135.2
889.0	44.8
896.0	58.1
903.0	122.9
910.0	58.1
917.0	52.4
924.0	115.2
931.0	54.3
938.0	61.0
945.0	123.8
952.0	51.4
959.0	49.5
966.0	118.1
973.0	52.4
980.0	61.0
987.0	99.0
994.0	49.5
1001.0	44.8
1008.0	96.2
1015.0	39.0
1022.0	44.8
1029.0	81.9
1036.0	30.5
1043.0	34.3
1050.0	78.1
1057.0	36.2

1064.0	29.5
1071.0	73.3
1078.0	25.7
1085.0	20.0
1092.0	67.6
1099.0	33.3
1106.0	25.7
1113.0	61.0
1120.0	18.1
1127.0	28.6
1134.0	42.9
1141.0	28.6
1148.0	14.3
1155.0	46.7
1162.0	21.0

3.2. Determining the number of stages and the amount of water required for solvent exchange

Table S3 Permeate flux v/s time data from diafiltration experiment with feed containing 5 wt% ethanol (rest water). Corresponds to the Figure 3 of the manuscript.

**Ethanol water system**							
**Stage 0**		**Stage 1**		**Stage 2**		**Stage 3**	
**Time**	**Flux**	**Time**	**Flux**	**Time**	**Flux**	**Time**	**Flux**
**s**	**kg (m² h)⁻¹**	**s**	**kg (m² h)⁻¹**	**s**	**kg (m² h)⁻¹**	**s**	**kg (m² h)⁻¹**
7.00	55.24	7.00	73.33	7.00	95.24	7.00	100.95
14.00	67.62	14.00	67.62	14.00	102.86	14.00	121.90
21.00	68.57	21.00	80.00	21.00	107.62	21.00	115.24
28.00	78.10	28.00	81.90	28.00	112.38	28.00	119.05
35.00	79.05	35.00	80.95	35.00	120.95	35.00	121.90
42.00	80.00	42.00	83.81	42.00	112.38	42.00	108.57
49.00	80.95	49.00	86.67	49.00	120.95	49.00	122.86
56.00	95.24	56.00	81.90	56.00	127.62	56.00	146.67
63.00	98.10	63.00	81.90	63.00	117.14	63.00	127.62
70.00	92.38	70.00	86.67	70.00	121.90	70.00	133.33

77.00	85.71	77.00	84.76	77.00	125.71	77.00	139.05
84.00	97.14	84.00	82.86	84.00	117.14	84.00	130.48
91.00	96.19	91.00	87.62	91.00	120.00	91.00	134.29
98.00	96.19	98.00	101.90	98.00	122.86	98.00	139.05
105.00	100.00	105.00	108.57	105.00	114.29	105.00	128.57
112.00	100.00	112.00	105.71	112.00	119.05	112.00	135.24
119.00	98.10	119.00	109.52	119.00	124.76	119.00	137.14
126.00	93.33	126.00	110.48	126.00	111.43	126.00	125.71
133.00	98.10	133.00	104.76	133.00	118.10	133.00	131.43
140.00	110.48	140.00	106.67	140.00	124.76	140.00	129.52
147.00	89.52	147.00	108.57	147.00	108.57	147.00	124.76
154.00	92.38	154.00	117.14	154.00	108.57	154.00	125.71
161.00	100.95	161.00	100.00	161.00	115.24	161.00	125.71
168.00	100.00	168.00	98.10	168.00	118.10	168.00	121.90
175.00	95.24	175.00	106.67	175.00	114.29	175.00	122.86
182.00	99.05	182.00	105.71	182.00	116.19	182.00	125.71
189.00	98.10	189.00	100.95	189.00	113.33	189.00	117.14
196.00	89.52	196.00	104.76	196.00	111.43	196.00	121.90
203.00	92.38	203.00	110.48	203.00	111.43	203.00	121.90
210.00	98.10	210.00	100.00	210.00	117.14	210.00	113.33
217.00	90.48	217.00	94.29	217.00	110.48	217.00	119.05
224.00	94.29	224.00	102.86	224.00	116.19	224.00	158.10
231.00	88.57	231.00	100.95	231.00	122.86	231.00	116.19
238.00	90.48	238.00	99.05	238.00	112.38	238.00	118.10
245.00	104.76	245.00	100.00	245.00	117.14	245.00	326.67
252.00	77.14	252.00	102.86	252.00	123.81	252.00	122.86
259.00	95.24	259.00	96.19	259.00	100.95	259.00	112.38
266.00	87.62	266.00	99.05	266.00	116.19	266.00	317.14
273.00	101.90	273.00	100.95	273.00	118.10	273.00	117.14
280.00	77.14	280.00	112.38	280.00	120.00	280.00	120.00
287.00	90.48	287.00	103.81	287.00	165.71	287.00	334.29
294.00	87.62	294.00	107.62	294.00	109.96	294.00	121.90
301.00	96.19	301.00	113.33	301.00	107.62	301.00	113.33
308.00	80.00	308.00	93.33	308.00	311.43	308.00	337.14
315.00	90.48	315.00	100.95	315.00	113.33	315.00	120.95
322.00	83.81	322.00	104.76	322.00	113.33	322.00	126.67
329.00	98.10	329.00	100.95	329.00	326.67	329.00	320.95
336.00	78.10	336.00	257.14	336.00	108.57	336.00	122.86
343.00	83.81	343.00	102.86	343.00	107.62	343.00	118.10
350.00	80.95	350.00	96.19	350.00	319.05	350.00	315.24
357.00	92.38	357.00	267.62	357.00	110.48	357.00	119.05
364.00	89.52	364.00	94.29	364.00	112.38	364.00	125.71
371.00	75.24	371.00	93.33	371.00	309.52	371.00	305.71
378.00	81.39	378.00	262.86	378.00	113.33	378.00	123.81
385.00	88.57	385.00	101.90	385.00	114.29	385.00	115.24
392.00	85.71	392.00	105.71	392.00	312.38	392.00	294.29
399.00	82.86	399.00	278.10	399.00	104.76	399.00	80.00
406.00	143.81	406.00	117.14	406.00	110.48	406.00	67.62
413.00	81.39	413.00	99.05	413.00	289.52	413.00	144.76

420.00	74.29	420.00	290.48	420.00	110.48		
427.00	184.76	427.00	100.00	427.00	114.29		
434.00	83.98	434.00	102.86	434.00	263.81		
441.00	71.43	441.00	277.14	441.00	97.14		
448.00	184.76	448.00	101.90	448.00	72.38		
455.00	87.62	455.00	106.67	455.00	215.24		
462.00	73.33	462.00	260.95	462.00	68.57		
469.00	180.95	469.00	108.57	469.00	56.19		
476.00	81.90	476.00	83.81	476.00	166.67		
483.00	82.86	483.00	265.71	483.00	52.38		
490.00	175.24	490.00	87.62	490.00	59.05		
497.00	83.81	497.00	89.52	497.00	116.19		
504.00	80.00	504.00	205.71				
511.00	167.62	511.00	52.38				
518.00	83.81	518.00	54.29				
525.00	80.95	525.00	120.00				
532.00	173.33						
539.00	76.19						
546.00	89.52						
553.00	165.71						
560.00	81.90						
567.00	75.24						
574.00	177.14						
581.00	72.38						
588.00	82.86						
595.00	171.43						
602.00	79.05						
609.00	65.71						
616.00	140.95						

Table S4 Permeate flux v/s time data for diafiltration with an aqueous feed containing 0.1 wt% model particles and 5 wt% ethanol (rest water). Corresponds to the Figure 3 of the manuscript.

**Ethanol water model particle system**							
**Stage 0**		**Stage 1**		**Stage 2**		**Stage 3**	
**Time**	**Flux**	**Time**	**Flux**	**Time**	**Flux**	**Time**	**Flux**
**s**	**kg (m^2 h)$^{-1}$**	**s**	**kg (m^2 h)$^{-1}$**	**s**	**kg (m^2 h)$^{-1}$**	**s**	**kg (m^2 h)$^{-1}$**
7.00	50.48	7.00	58.10	7.00	70.48	7.00	90.48
14.00	49.52	14.00	64.76	14.00	82.86	14.00	93.33
21.00	57.14	21.00	75.24	21.00	91.43	21.00	96.19
28.00	67.62	28.00	78.10	28.00	77.14	28.00	96.19
35.00	66.67	35.00	80.00	35.00	88.57	35.00	99.05
42.00	57.14	42.00	67.62	42.00	84.76	42.00	104.76
49.00	65.71	49.00	80.95	49.00	105.71	49.00	93.33
56.00	55.24	56.00	73.33	56.00	78.10	56.00	97.14
63.00	71.43	63.00	80.95	63.00	94.29	63.00	95.24
70.00	60.95	70.00	76.19	70.00	86.67	70.00	102.86
77.00	73.33	77.00	80.95	77.00	100.95	77.00	94.29

84.00	67.62	84.00	81.90	84.00	80.95	84.00	87.62
91.00	58.10	91.00	72.38	91.00	91.43	91.00	98.10
98.00	70.48	98.00	86.67	98.00	92.38	98.00	97.14
105.00	62.86	105.00	68.57	105.00	98.10	105.00	92.38
112.00	75.24	112.00	81.90	112.00	79.05	112.00	96.19
119.00	70.48	119.00	72.38	119.00	92.38	119.00	91.43
126.00	60.00	126.00	81.90	126.00	91.43	126.00	91.43
133.00	62.86	133.00	75.24	133.00	98.10	133.00	90.48
140.00	70.48	140.00	79.05	140.00	78.10	140.00	94.29
147.00	59.05	147.00	79.05	147.00	91.43	147.00	93.33
154.00	72.38	154.00	72.38	154.00	91.43	154.00	92.38
161.00	64.76	161.00	73.33	161.00	95.24	161.00	88.57
168.00	69.52	168.00	80.00	168.00	78.10	168.00	99.05
175.00	64.76	175.00	70.48	175.00	90.48	175.00	108.57
182.00	66.67	182.00	80.95	182.00	90.48	182.00	86.67
189.00	59.05	189.00	79.05	189.00	95.24	189.00	102.86
196.00	68.57	196.00	76.19	196.00	78.10	196.00	102.86
203.00	66.67	203.00	77.14	203.00	90.48	203.00	104.76
210.00	60.00	210.00	72.38	210.00	85.71	210.00	99.05
217.00	60.95	217.00	75.24	217.00	98.10	217.00	104.76
224.00	71.43	224.00	74.29	224.00	77.14	224.00	104.76
231.00	68.57	231.00	71.43	231.00	89.52	231.00	100.95
238.00	57.14	238.00	77.14	238.00	86.67	238.00	102.86
245.00	61.90	245.00	77.14	245.00	96.19	245.00	100.00
252.00	67.62	252.00	70.48	252.00	78.10	252.00	108.57
259.00	56.19	259.00	82.86	259.00	86.67	259.00	99.05
266.00	61.90	266.00	67.62	266.00	83.81	266.00	105.71
273.00	68.57	273.00	74.29	273.00	86.67	273.00	101.90
280.00	66.67	280.00	78.10	280.00	87.62	280.00	100.95
287.00	57.14	287.00	78.10	287.00	81.90	287.00	97.14
294.00	64.76	294.00	74.29	294.00	77.92	294.00	103.81
301.00	65.71	301.00	76.19	301.00	89.52	301.00	108.57
308.00	67.62	308.00	69.52	308.00	86.67	308.00	100.00
315.00	54.29	315.00	75.24	315.00	87.62	315.00	101.90
322.00	86.67	322.00	77.14	322.00	93.33	322.00	91.43
329.00	56.19	329.00	73.33	329.00	132.38	329.00	139.05
336.00	68.57	336.00	95.24	336.00	88.57	336.00	102.86
343.00	166.67	343.00	78.10	343.00	86.67	343.00	101.90
350.00	59.05	350.00	79.05	350.00	254.29	350.00	265.71
357.00	60.95	357.00	201.90	357.00	95.24	357.00	99.05
364.00	169.52	364.00	77.14	364.00	86.67	364.00	98.10
371.00	62.86	371.00	78.10	371.00	250.48	371.00	257.14
378.00	52.81	378.00	203.81	378.00	91.43	378.00	94.29
385.00	172.38	385.00	73.33	385.00	91.43	385.00	91.43
392.00	60.00	392.00	80.00	392.00	253.33	392.00	262.86
399.00	63.81	399.00	202.86	399.00	92.38	399.00	86.67
406.00	169.52	406.00	73.33	406.00	93.33	406.00	95.24
413.00	53.68	413.00	77.14	413.00	250.48	413.00	255.24
420.00	62.86	420.00	200.00	420.00	90.48	420.00	84.76

427.00	158.10	427.00	71.43	427.00	85.71	427.00	92.38
434.00	60.61	434.00	76.19	434.00	256.19	434.00	254.29
441.00	55.24	441.00	209.52	441.00	90.48	441.00	93.33
448.00	167.62	448.00	69.52	448.00	89.52	448.00	99.05
455.00	54.29	455.00	79.05	455.00	232.38	455.00	243.81
462.00	66.67	462.00	202.86	462.00	94.29	462.00	104.76
469.00	167.62	469.00	70.48	469.00	84.76	469.00	80.95
476.00	56.19	476.00	79.05	476.00	248.57	476.00	278.10
483.00	57.14	483.00	205.71	483.00	87.62	483.00	81.90
490.00	172.38	490.00	73.33	490.00	91.43	490.00	97.14
497.00	57.14	497.00	75.24	497.00	248.57	497.00	277.14
504.00	58.10	504.00	204.76	504.00	91.43	504.00	87.62
511.00	155.24	511.00	70.48	511.00	82.86	511.00	90.48
518.00	67.62	518.00	78.10	518.00	243.81	518.00	207.62
525.00	50.48	525.00	200.00	525.00	91.43	525.00	64.76
532.00	163.81	532.00	71.43	532.00	85.71	532.00	53.33
539.00	56.19	539.00	76.19	539.00	239.05		
546.00	68.57	546.00	203.81	546.00	62.86		
553.00	160.00	553.00	73.33	553.00	65.71		
560.00	56.19	560.00	73.33	560.00	158.10		
567.00	54.29	567.00	205.71	567.00	49.52		
574.00	168.57	574.00	76.19	574.00	47.62		
581.00	56.19	581.00	73.33	581.00	102.86		
588.00	59.05	588.00	202.86				
595.00	163.81	595.00	70.48				
602.00	61.90	602.00	79.05				
609.00	54.29	609.00	198.10				
616.00	155.24	616.00	69.52				
623.00	64.76	623.00	73.33				
630.00	51.43	630.00	174.29				
637.00	163.81	637.00	60.95				
644.00	52.38	644.00	48.57				
651.00	70.48	651.00	131.43				
658.00	152.38	658.00	40.95				
665.00	60.00	665.00	38.10				
672.00	56.19	672.00	92.38				
679.00	165.71	679.00	31.43				
686.00	50.48	686.00	28.57				
693.00	54.29	693.00	71.43				
700.00	166.67						
707.00	53.33						
714.00	60.95						
721.00	156.19						
728.00	62.86						
735.00	52.38						
742.00	139.05						
749.00	50.48						
756.00	38.10						
763.00	104.76						

Table S5 Permeate flux profile for the diafiltration with an aqueous feed containing: (1) 0.1 wt% HPMC E3 solution (in water), (2) 0.1 wt% model particles and 0.1 wt% HPMC E3 in water, (3) 5 wt% ethanol solution and (4) an aqueous feed containing 0.1 wt% model particles and 5 wt% ethanol. Corresponds to Figure 4 in the manuscript.

Time	Flux	Flux	Flux	Flux
s	kg (m² h)⁻¹ HPMC in Water	kg (m² h)⁻¹ HPMC + Model particles in Water	kg (m² h)⁻¹ Ethanol / Water	kg (m² h)⁻¹ Model particles in Ethanol / Water
0.00				
0.17	64.22	53.52	96.00	75.00
0.33	63.14	50.32	96.00	75.00
0.50	59.86	46.63	96.00	75.00
0.67	57.25	43.94	96.00	75.00
0.83	55.14	41.70	96.00	75.00
1.00	53.66	39.99	96.00	75.00
1.17	52.34	38.41	96.00	75.00
1.33	51.23	37.14	96.00	75.00
1.50	50.11	35.91	96.00	75.00
1.67	49.16	34.89	96.00	75.00
1.83	48.34	33.82	96.00	75.00
2.00	47.71	32.78	96.00	75.00
2.17	47.02	32.16	96.00	75.00
2.33	46.45	31.36	96.00	75.00
2.50	45.90	30.67	96.00	75.00
2.67	45.38	30.00	96.00	75.00
2.83	44.90	29.40	96.00	75.00
3.00	44.49	28.89	96.00	75.00
3.17	44.06	28.29	96.00	75.00
3.33	43.60	27.65	96.00	75.00
3.50	43.39	27.11	96.00	75.00
3.67	42.81	26.73	96.00	75.00
3.83	42.61	26.27	96.00	75.00
4.00	42.17	25.89	96.00	75.00
4.17	41.91	25.38	96.00	75.00
4.33	41.62	24.98	96.00	75.00
4.50	41.21	24.65	96.00	75.00
4.67	40.89	24.25	96.00	75.00
4.83	40.70	23.85	96.00	75.00
5.00	40.40	23.65	96.00	75.00
5.17	40.13	23.31	96.00	75.00
5.33	39.78	23.00	96.00	75.00
5.50	39.54	22.67	96.00	75.00
5.67	39.33	22.40	96.00	75.00
5.83	39.13	22.05	96.00	75.00
6.00	38.69	21.87	96.00	75.00

6.17	38.44	21.60	96.00	75.00
6.33	38.24	21.18	96.00	75.00
6.50	38.07	20.99	96.00	75.00
6.67	37.84	20.72	96.00	75.00
6.83	37.69	20.70	96.00	75.00
7.00	37.50	20.48	96.00	75.00
7.17	37.27	20.26	96.00	75.00
7.33	37.11	20.03	96.00	75.00
7.50	36.81	19.92	96.00	75.00
7.67	36.61	19.68	96.00	75.00
7.83	36.45	19.50	96.00	75.00
8.00	36.31	19.26	96.00	75.00
8.17	36.07	19.12	96.00	75.00
8.33	35.84	19.10	96.00	75.00
8.50	35.50	19.04	96.00	75.00
8.67	35.46	18.88	96.00	75.00
8.83	35.19	18.70	96.00	75.00
9.00	35.16	18.59	96.00	75.00
9.17	34.88	18.44	96.00	75.00
9.33	34.76	18.35	96.00	75.00
9.50	34.53	18.24	96.00	75.00
9.67	34.43	18.10	96.00	75.00
9.83	34.21	18.01	96.00	75.00
10.00	34.15	17.81	96.00	75.00
10.17	33.92	17.61	96.00	75.00
10.33	33.81	17.57	96.00	75.00
10.50	33.68	17.46	96.00	75.00
10.67	33.52	17.30	96.00	75.00
10.83	33.59	17.30	96.00	75.00
11.00	33.51	17.12	96.00	75.00
11.17	33.31	17.04	96.00	75.00
11.33	33.05	16.99	96.00	75.00
11.50	32.91	16.93	96.00	75.00
11.67	32.83	16.77	96.00	75.00
11.83	32.75	16.73	96.00	75.00
12.00	32.52	16.57	96.00	75.00
12.17	32.20	16.48	96.00	75.00
12.33	32.16	16.41	96.00	75.00
12.50	32.12	16.30	96.00	75.00
12.67	32.10	16.33	96.00	75.00
12.83	31.96	16.17	96.00	75.00
13.00	31.66	16.08	96.00	75.00
13.17	31.61	15.97	96.00	75.00
13.33	31.64	15.95	96.00	75.00
13.50	31.63	15.77	96.00	75.00
13.67	31.44	15.77	96.00	75.00
13.83	31.33	15.66	96.00	75.00
14.00	31.30	15.55	96.00	75.00
14.17	31.23	15.48	96.00	75.00

14.33	31.07	15.46	96.00	75.00
14.50	31.00	15.35	96.00	75.00
14.67	30.96	15.24	96.00	75.00
14.83	30.88	15.10	96.00	75.00
15.00	30.68	15.15	96.00	75.00
15.17	30.63	15.15	96.00	75.00
15.33	30.64	15.06	96.00	75.00
15.50	30.53	15.10	96.00	75.00
15.67	30.57	14.93	96.00	75.00
15.83	30.30	14.84	96.00	75.00
16.00	30.32	14.72	96.00	75.00
16.17	30.29	14.68	96.00	75.00
16.33	30.25	14.59	96.00	75.00
16.50	30.13	14.50	96.00	75.00
16.67	30.11	14.54	96.00	75.00
16.83	30.08	14.32	96.00	75.00
17.00	29.89	14.48	96.00	75.00

3.4. Case study: Removing ethanol from a naproxen crystal suspension after anti-solvent crystallization

Table S6 Permeate flux profile over time during discontinuous and semi-continuous diafiltration of naproxen crystals (0.2 wt%) suspended in a solution of 0.1 wt% HPMC E3, 3.8 wt% ethanol and 95.9 wt% water. The suspension used in both cases had the same composition. The data corresponds to the figure 5 of the manuscript.

**Time**	**Flux**	
**s**	**kg (m² h)⁻¹**	
	**Discontinuous**	**Semi-continuous**
0.17	39.70	40.55
0.50	37.28	38.32
0.83	34.48	35.50
1.17	32.44	33.97
1.50	30.92	32.76
1.83	29.68	31.72
2.17	28.61	30.80
2.50	27.75	29.92
2.83	27.06	29.29
3.17	26.48	28.70
3.50	25.88	28.17
3.83	25.33	27.72
4.17	25.02	27.12
4.50	24.57	26.65
4.83	24.30	26.23
5.17	23.82	25.68
5.50	23.44	25.27
5.83	23.08	25.01
6.17	22.64	24.67

6.50	22.24	24.36
6.83	21.88	24.08
7.17	21.50	23.92
7.50	21.13	23.66
7.83	20.77	23.33
8.17	20.39	23.02
8.50	20.11	22.93
8.83	19.84	22.68
9.17	19.39	22.50
9.50	19.04	22.28
9.83	18.68	21.93
10.17	18.51	21.91
10.50	18.28	21.64
10.83	18.00	21.47
11.17	17.86	21.33
11.50	17.71	21.00
11.83	17.53	21.00
12.17	17.42	20.64
12.50	17.31	20.82
12.83	17.02	20.65
13.17	16.99	20.49
13.50	16.77	20.05
13.83	16.59	19.85
14.17	16.48	19.58
14.50	16.42	19.37
14.83	16.26	19.13
15.17	16.11	18.97
15.50	16.00	18.58
15.83	15.91	18.10
16.17	15.77	18.15
16.50	15.60	18.16
16.83	15.57	17.83
17.17	15.40	17.61
17.50	15.31	17.25
17.83	15.31	16.96
18.17	15.02	17.25
18.50	14.82	17.10
18.83	37.59	16.99
19.17	35.50	16.75
19.50	33.30	16.81
19.83	31.88	16.77
20.17	30.59	16.66
20.50	29.52	16.67
20.83	28.48	16.62
21.17	27.61	16.58
21.50	26.86	16.44
21.83	26.35	16.21
22.17	25.68	16.44
22.50	25.15	16.36

22.83	24.64	16.44
23.17	24.24	15.93
23.50	23.79	16.09
23.83	23.33	16.09
24.17	22.99	16.23
24.50	22.68	15.97
24.83	22.30	15.88
25.17	21.99	15.79
25.50	21.68	15.50
25.83	21.39	15.70
26.17	21.17	15.67
26.50	20.88	15.32
26.83	20.62	15.45
27.17	20.35	15.44
27.50	20.13	15.37
27.83	19.93	15.72
28.17	19.64	15.68
28.50	19.55	15.74
28.83	19.39	15.61
29.17	19.10	15.72
29.50	18.88	16.13
29.83	18.66	16.47
30.17	18.57	16.56
30.50	18.42	17.08
30.83	18.35	16.88
31.17	18.11	16.92
31.50	17.97	17.25
31.83	17.84	17.28
32.17	17.68	17.29
32.50	17.46	17.72
32.83	17.42	17.21
33.17	17.37	17.47
33.50	17.13	17.33
33.83	17.02	17.83
34.17	17.00	17.79
34.50	16.97	17.50
34.83	16.79	17.26
35.17	16.59	17.24
35.50	16.53	17.32
35.83	16.37	17.61
36.17	16.33	17.23
36.50	16.26	17.25
36.83	15.86	17.33
37.17	15.86	17.41
37.50	39.08	16.97
37.83	38.10	17.21
38.17	35.59	17.07
38.50	33.92	17.09
38.83	32.52	16.65

39.17	31.21	16.75
39.50	30.19	16.77
39.83	29.17	17.18
40.17	28.41	16.76
40.50	27.52	16.83
40.83	26.92	16.94
41.17	26.32	17.10
41.50	25.86	16.79
41.83	25.30	17.07
42.17	24.88	16.87
42.50	24.44	17.13
42.83	24.08	16.96
43.17	23.82	16.79
43.50	23.31	16.93
43.83	22.95	17.06
44.17	22.71	17.02
44.50	22.33	17.02
44.83	22.17	16.63
45.17	21.77	17.22
45.50	21.68	16.95
45.83	21.35	16.75
46.17	21.13	16.69
46.50	20.84	17.10
46.83	20.68	16.67
47.17	20.44	16.67
47.50	20.33	16.54
47.83	19.95	16.68
48.17	19.93	16.84
48.50	19.77	16.79
48.83	19.55	16.82
49.17	19.35	16.70
49.50	19.26	16.74
49.83	19.06	16.72
50.17	18.86	16.59
50.50	18.75	16.63
50.83	18.60	16.58
51.17	18.39	16.62
51.50	18.42	16.61
51.83	18.22	16.64
52.17	17.93	16.71
52.50	17.75	16.69
52.83	17.68	16.58
53.17	17.22	16.68
53.50	17.13	16.61
53.83	17.20	16.49
54.17	17.20	16.60
54.50	17.00	16.49
54.83	16.80	16.56
55.17	16.73	16.42

55.50	39.52	16.48
55.83	37.65	16.46
56.17	35.41	16.48
56.50	33.61	16.45
56.83	32.21	16.47
57.17	31.10	16.45
57.50	30.08	16.29
57.83	29.12	16.41
58.17	28.50	16.34
58.50	27.77	16.53
58.83	27.13	16.39
59.17	26.59	16.32
59.50	26.06	16.37
59.83	25.62	16.41
60.17	25.22	16.55
60.50	24.90	16.45
60.83	24.37	16.40
61.17	24.13	16.35
61.50	23.84	16.47
61.83	23.50	16.41
62.17	23.17	16.37
62.50	22.88	16.41
62.83	22.57	16.45
63.17	22.13	16.45
63.50	21.95	16.32
63.83	21.77	16.40
64.17	21.48	16.37
64.50	21.28	16.42
64.83	20.99	16.27
65.17	20.93	16.26
65.50	20.64	16.38
65.83	20.66	16.29
66.17	20.39	16.33
66.50	20.11	16.22
66.83	19.57	16.28
67.17	19.31	16.34
67.50	19.19	16.29
67.83	19.13	16.19
68.17	19.13	16.16
68.50	18.71	16.16
68.83	18.84	16.20
69.17	18.53	16.22
69.50	18.39	16.17
69.83	18.44	16.16
70.17	18.11	16.11
70.50	17.88	15.98
70.83	17.82	16.06
71.17	17.71	16.09
71.50	17.46	16.03

71.83	17.82	16.01
72.17	17.77	15.87
72.50		15.97
72.83		16.01
73.17		16.03
73.50		15.90
73.83		16.00
74.17		16.01
74.50		15.92
74.83		15.84
75.17		15.94
75.50		15.99
75.83		16.03
76.17		15.83
76.50		15.81
76.83		15.78
77.17		15.91
77.50		15.94
77.83		15.89
78.17		15.82
78.50		15.95
78.83		15.79
79.17		15.83
79.50		15.91
79.83		15.91
80.17		15.82
80.50		15.80
80.83		15.71
81.17		15.83
81.50		15.89
81.83		15.86
82.17		15.92
82.50		15.72
82.83		15.76
83.17		15.68
83.50		15.71
83.83		15.74
84.17		15.68
84.50		15.71
84.83		15.76
85.17		15.71
85.50		15.67
85.83		15.73
86.17		15.67
86.50		15.63
43.50		16.93
43.67		17.22
43.83		17.06
44.00		17.22

44.17	17.02
44.33	16.90
44.50	17.02
44.67	16.70
44.83	16.63
45.00	16.77
45.17	17.22
45.33	17.09
45.50	16.95
45.67	16.89
45.83	16.75
46.00	17.12
46.17	16.69
46.33	16.77
46.50	17.10
46.67	16.83
46.83	16.67
47.00	16.45
47.17	16.67
47.33	16.68
47.50	16.54
47.67	16.62
47.83	16.68
48.00	16.75
48.17	16.84
48.33	16.84
48.50	16.79
48.67	16.63
48.83	16.82
49.00	16.63
49.17	16.70
49.33	16.76
49.50	16.74
49.67	16.80
49.83	16.72
50.00	16.70
50.17	16.59
50.33	16.63
50.50	16.63
50.67	16.56
50.83	16.58
51.00	16.58
51.17	16.62
51.33	16.64
51.50	16.61
51.67	16.69
51.83	16.64
52.00	16.81
52.17	16.71

52.33	16.64
52.50	16.69
52.67	16.62
52.83	16.58
53.00	16.61
53.17	16.68
53.33	16.53
53.50	16.61
53.67	16.58
53.83	16.49
54.00	16.53
54.17	16.60
54.33	16.48
54.50	16.49
54.67	16.51
54.83	16.56
55.00	16.40
55.17	16.42
55.33	16.52
55.50	16.48
55.67	16.56
55.83	16.46
56.00	16.48
56.17	16.48
56.33	16.41
56.50	16.45
56.67	16.48
56.83	16.47
57.00	16.43
57.17	16.45
57.33	16.44
57.50	16.29
57.67	16.45
57.83	16.41
58.00	16.45
58.17	16.34
58.33	16.46
58.50	16.53
58.67	16.46
58.83	16.39
59.00	16.34
59.17	16.32
59.33	16.38
59.50	16.37
59.67	16.44
59.83	16.41
60.00	16.55
60.17	16.55
60.33	16.43

60.50	16.45
60.67	16.37
60.83	16.40
61.00	16.37
61.17	16.35
61.33	16.46
61.50	16.47
61.67	16.47
61.83	16.41
62.00	16.36
62.17	16.37
62.33	16.41
62.50	16.41
62.67	16.39
62.83	16.45
63.00	16.40
63.17	16.45
63.33	16.45
63.50	16.32
63.67	16.34
63.83	16.40
64.00	16.50
64.17	16.37
64.33	16.36
64.50	16.42
64.67	16.18
64.83	16.27
65.00	16.28
65.17	16.26
65.33	16.35
65.50	16.38
65.67	16.28
65.83	16.29
66.00	16.34
66.17	16.33
66.33	16.29
66.50	16.22
66.67	16.37
66.83	16.28
67.00	16.30
67.17	16.34
67.33	16.29
67.50	16.29
67.67	16.21
67.83	16.19
68.00	16.21
68.17	16.16
68.33	16.20
68.50	16.16

68.67	16.12
68.83	16.20
69.00	16.08
69.17	16.22
69.33	16.24
69.50	16.17
69.67	16.22
69.83	16.16
70.00	16.17
70.17	16.11
70.33	16.03
70.50	15.98
70.67	16.11
70.83	16.06
71.00	16.07
71.17	16.09
71.33	16.00
71.50	16.03
71.67	15.88
71.83	16.01
72.00	15.99
72.17	15.87
72.33	15.94
72.50	15.97
72.67	16.00
72.83	16.01
73.00	16.00
73.17	16.03
73.33	16.00
73.50	15.90
73.67	16.02
73.83	16.00
74.00	15.99
74.17	16.01
74.33	15.96
74.50	15.92
74.67	15.93
74.83	15.84
75.00	15.89
75.17	15.94
75.33	15.95
75.50	15.99
75.67	15.92
75.83	16.03
76.00	15.95
76.17	15.83
76.33	15.83
76.50	15.81
76.67	15.88

76.83	15.78
77.00	15.91
77.17	15.91
77.33	15.82
77.50	15.94
77.67	15.88
77.83	15.89
78.00	15.91
78.17	15.82
78.33	15.82
78.50	15.95
78.67	15.87
78.83	15.79
79.00	15.92
79.17	15.83
79.33	15.81
79.50	15.91
79.67	15.79
79.83	15.91
80.00	15.83
80.17	15.82
80.33	15.77
80.50	15.80
80.67	15.84
80.83	15.71
81.00	15.77
81.17	15.83
81.33	15.85
81.50	15.89
81.67	15.79
81.83	15.86
82.00	15.79
82.17	15.92
82.33	15.92
82.50	15.72
82.67	15.79
82.83	15.76
83.00	15.72
83.17	15.68
83.33	15.75
83.50	15.71
83.67	15.67
83.83	15.74
84.00	15.61
84.17	15.68
84.33	15.72
84.50	15.71
84.67	15.77
84.83	15.76

85.00	15.75
85.17	15.71
85.33	15.80
85.50	15.67
85.67	15.66
85.83	15.73
86.00	15.68
86.17	15.67
86.33	15.67
86.50	15.63
